# Automated assessment of 3D facial asymmetry: a systematic review

**DOI:** 10.1093/ejo/cjag012

**Published:** 2026-05-26

**Authors:** Jiale Peng, Wanting Qu, Shiming Zhang, Yifan Lin

**Affiliations:** Division of Paediatric Dentistry and Orthodontics, Faculty of Dentistry, The University of Hong Kong, Hong Kong SAR, China; Division of Paediatric Dentistry and Orthodontics, Faculty of Dentistry, The University of Hong Kong, Hong Kong SAR, China; Department of Electrical and Electronic Engineering, The University of Hong Kong, Hong Kong SAR, China; State Key Laboratory of Pharmaceutical Biotechnology, The University of Hong Kong, Hong Kong SAR, China; Division of Paediatric Dentistry and Orthodontics, Faculty of Dentistry, The University of Hong Kong, Hong Kong SAR, China

**Keywords:** facial asymmetry, automated, algorithm, deep learning, three-dimensional facial scan

## Abstract

**Objectives:**

This systematic review aims to evaluate the clinical applicability of automated 3D facial asymmetry assessment methods based on 3D facial scans.

**Search Methods:**

A comprehensive search of electronic databases (PubMed, Web of Science, EMBASE, Medline, and Scopus) and manual literature searches were conducted in April 2025.

**Selection Criteria:**

Studies that were published in English and evaluated the validity or reliability of automated asymmetry assessment methods in 3D facial scans for medical or biological settings were included.

**Data Collection and Analysis:**

Two reviewers independently screened the articles for eligibility. Risk of bias was assessed using QUADAS-2, and the certainty of evidence was graded using the Grading of Recommendations Assessment, Development, and Evaluation framework.

**Results:**

Fourteen studies met the inclusion criteria and were analyzed for methodology, validity, and reliability. Methodologies for assessing facial asymmetry were categorized into four approaches: landmark-based, depth-stratified, original-mirror alignment, and template-based approaches. While landmark and depth-stratified methods rely on sparse data, original-mirror and template-based methods enable comprehensive surface analysis. Six studies evaluating validity against alternative methods, synthetic ground truth, or human ratings consistently demonstrated moderate-to-strong correlation coefficients and classification accuracy. Reliability was examined across nine studies using repeated measurements and multi-observer designs, generally showing minimal measurement variation. Notably, methodological analysis revealed that original-mirror alignment, typically implemented using unconstrained iterative closest point (ICP)-based best-fit registration, is susceptible to registration errors (the “Pinocchio effect”) in cases of severe asymmetry, whereas template-based methods mitigate this through correspondence transfer and weighted registration strategies that stabilize anatomical alignment.

**Limitations:**

The review is limited by a high risk of bias in primary studies and significant methodological heterogeneity.

**Conclusions:**

Despite “very low” certainty evidence, template-based approaches that transfer anatomical correspondence and apply weighted registration appear preferable due to their robustness against localized deformities. Conversely, original-mirror alignment, typically implemented via unconstrained ICP, remains a practical alternative for mild asymmetry. Future research should prioritize end-to-end deep learning automation, dynamic analysis, and the development of accessible, open-source tools to bridge the gap between technical innovation and routine clinical practice.

**Registration:**

PROSPERO (CRD420251025105).

## Introduction

Facial asymmetry, characterized by differences in the shape, size, or position between the two sides of the face, plays a significant role in aesthetics, attractiveness, and the functional integrity of the orofacial complex [1, 2]. For decades, the primary goals of orthodontic treatment have extended beyond achieving a stable occlusion to encompass facial balance and harmony [3, 4].

Despite this emphasis, minor soft tissue asymmetries may be overlooked during initial consultations by both doctors and patients. If subsequently detected during or after orthodontic treatment, such oversights can significantly increase the risk of doctor-patient conflicts [5]. Severe soft tissue asymmetries often necessitate combined orthodontic-orthognathic treatment, where the efficacy of surgical planning relies on accurate localization and quantification of the asymmetry [2, 6]. Consequently, precise assessment of soft tissue asymmetries is essential not only for formulating optimal individual treatment plans but also for facilitating effective patient communication [5].

Advancements in dental technology offer potential solutions, automating time-consuming tasks like tooth segmentation and landmark identification [7, 8]. Similarly, automated analysis of facial asymmetry promises to reduce intra- and inter-observer variability and processing time compared with manual methods [9]. However, the clinical applicability of automated facial asymmetry assessments remains subjects of debate [9, 10]. To the best of our knowledge, there exists a gap in the literature concerning the systematic evaluation of automated methods for quantifying 3D facial asymmetry using 3D facial scans.

Therefore, this systematic review aims to critically evaluate the clinical applicability of current methods for the automated evaluation of 3D facial asymmetry via facial scans. The findings are expected to offer clinicians evidence-based support for choosing appropriate assessment methods in practice, while helping researchers identify existing knowledge gaps and facilitate the development of a more robust automated assessment pipeline for facial asymmetry.

## Materials and methods

This systematic review adhered to the Preferred Reporting Items for Systematic Reviews and Meta-Analyses (PRISMA) guidelines [11] (Supplementary Table S1). After a full consensus among the authors, the review protocol was registered in the PROSPERO database (CRD420251025105).

### Eligibility criteria

Eligibility criteria were defined using the PICOS framework:

- Population: human subjects with minor or severe facial asymmetry.
- Intervention: automated evaluation of facial asymmetry using 3D facial scans.
- Comparison: methodological comparison, alongside reported validation against reference standards such as clinical expert assessments, alternative methods, or synthetic asymmetric patterns.
- Outcomes: Primary outcomes included the methodologies used for automated assessment and their key characteristics affecting clinical applicability. Secondary outcomes were quantitative performance metrics, such as reliability and validity (e.g. correlation with reference standards).
- Study design: clinical trials, prospective and retrospective cohort studies, comparative studies, validation or evaluation studies, and epidemiologic studies within the medical and biological fields.

Additionally, studies were required to report some form of validation process for their validity or reliability assessments to be included, to ensure the robustness of the evidence base.

The exclusion criteria were as follows:

- Studies utilizing 3D facial analysis solely for non-biological or medical purposes (e.g. forensic facial recognition, biometric security, computer vision tasks).
- Case reports, literature reviews, conference abstracts, and animal studies.
- Studies primarily focused on developing methodologies for constructing the mid-sagittal plane (MSP) without evaluating facial asymmetry.

Thus, the overall study objective based on the PICOS format was as follows: what is the most optimal method for the automated assessment of facial asymmetry via 3D facial scans within the medical and biological fields?

### Information sources and search strategy

Two independent reviewers (J.P. and W.Q.) systematically searched the following electronic databases from inception until April 2025: PubMed, Web of Science, EMBASE (via Ovid), Medline (via Ovid), Scopus. A comprehensive three-pronged strategy was implemented. This strategy combined search terms for the techniques of interest (automated assessment) with terms describing the diagnostic target (facial asymmetry via 3D facial scans), using the Boolean operator “AND.” Each concept consisted of MeSH terms and keywords. The full search strategy was presented in Supplementary Table S2. The search was restricted to studies involving human subjects and published in the English language; no date restrictions were applied beyond the April 2025 cutoff. Manual searches of reference lists and citations from relevant key articles supplemented the electronic search.

### Study selection and data extraction

A reference manager software, Endnote™, version X9 (Clarivate Analytics, Philadelphia, USA) was used for removal of duplicates and further selection. Two reviewers (J.P. and W.Q.) independently screened the titles and abstracts of remaining citations against the eligibility criteria. Articles deemed potentially eligible underwent full-text assessment by the same reviewers. The Cohen’s kappa value (*κ*) was used to determine the inter-reviewer agreement level. Any disagreement regarding inclusion or exclusion at either the abstract or full-text stage was resolved through discussion; unresolved conflicts were adjudicated by a third, experienced reviewer (Y.L.). If multiple publications from the same research group describe identical asymmetry evaluation methods, the publication offering the most comprehensive explanation of the methodology was selected.

One reviewer extracted data from the final included studies using a standardized form, which was then verified for validity and completeness by the second reviewer. Extracted data encompassed bibliographic information (title, authors, publication year), study characteristics (primary aim, dataset source, total sample size, sizes of subsets if applicable), technical details (3D image technique, software used for analysis, image preprocessing), algorithmic aspects (core algorithm), and outcomes (specific asymmetry assessment method evaluated, performance metrics reported for validity and reliability). Corresponding authors of included studies were contacted to request missing or supplementary data.

### Risk of bias assessment

The two reviewers assessed the risk of bias and applicability of the included studies independently using a tailored version of the Quality Assessment Tool for Diagnostic Accuracy Studies-2 (QUADAS-2) [12]. This tool evaluates four key domains: Patient Selection, Index Test, Reference Standard, and Flow and Timing. The first three domains were also assessed for applicability concerns. Risk of bias and applicability within each domain were rated as low, high, or unclear.

### Certainty of evidence

The certainty of evidence regarding the clinical applicability of various methodologies for automated facial asymmetry assessment using 3D scans was evaluated with the Grading of Recommendations Assessment, Development, and Evaluation (GRADE) scale [13]. Using this approach, each of the five domains (study design, risk of bias, inconsistency, indirectness, and imprecision) was assessed and classified as having no serious, serious, or very serious concerns. The overall evidence for each method was then graded as high, moderate, low, or very low.

### Synthesis of results

Given the considerable heterogeneity in methodology between the selected studies in terms of study design, sample, assessment methodology, reference standards, and performance metrics reported for validity and reliability, a qualitative synthesis was performed to identify and summarize the main findings of the included articles.

## Results

### Study selections

**Figure 1:**
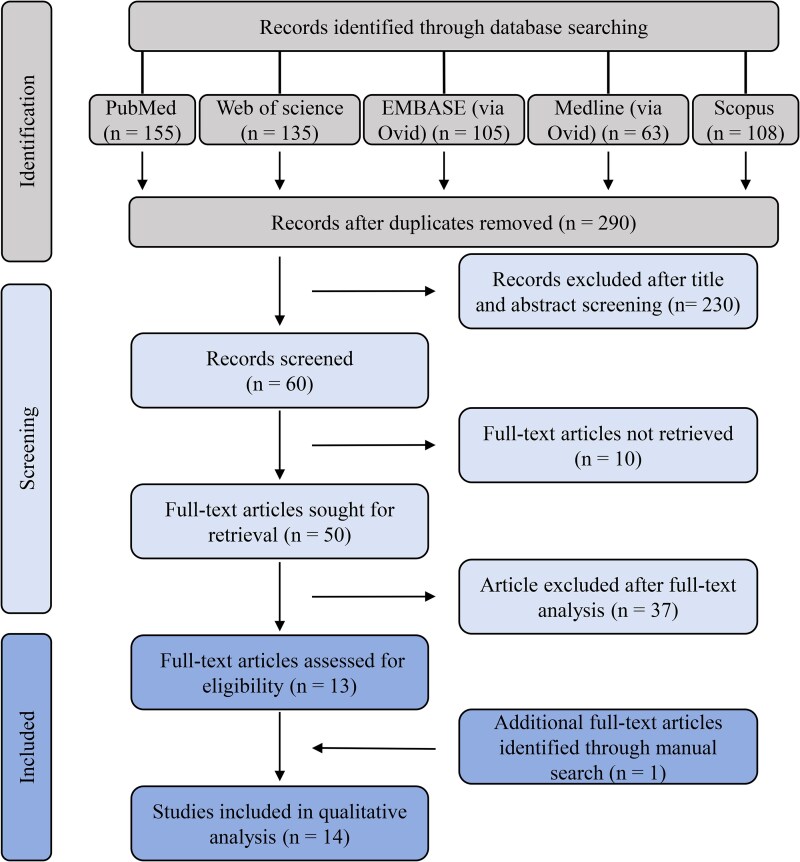
PRISMA flow chart illustrating the study selection process.

The study selection process is detailed in the PRISMA flow diagram (Fig. 1). Following searches across 5 databases, 290 manuscripts were initially identified after duplicate removal. Subsequent title and abstract screening excluded 230 studies, leaving 60 articles for full-text retrieval. During the screening process, reviewers initially disagreed on 14 of the 290 records. All discrepancies (8 in which Reviewer J.P. included a study that Reviewer W.Q. excluded, and 6 with the opposite discrepancy) were resolved through discussion with a third reviewer. The high level of inter-rater agreement (Cohen’s *κ* = 0.86) supports the robustness of the screening process. Full texts could not be obtained for 10 studies (primarily conference posters), resulting in 50 articles undergoing a detailed full-text assessment against the eligibility criteria. Of these, 13 studies met the inclusion criteria and were selected for qualitative synthesis. Excluded studies and their reasons are summarized in Supplementary Table S3. An additional study was identified through manual reference searching, yielding 14 studies for final inclusion [14–27]. Among these, five studies exclusively reported methodological validity [14, 18, 19, 21, 22]. One study addressed both validity and reliability [27], and eight studies focused solely on reliability [15–17, 20, 23–26 . Substantial methodological heterogeneity and divergent outcome measures precluded quantitative synthesis (meta-analysis).

### Study quality assessment

A tailored QUADAS-2 tool assessed methodological quality (Tables 1 and S4). In the domain of patient selection, eight studies demonstrated a high or unclear risk of bias, with two unclear applicability concerns; this issue stemmed from the use of non-consecutive or non-random participant sampling and insufficient characterization of the study population [15–18, 20–22, 24]. Regarding the index test, five studies were deemed to have a high or unclear risk of bias arising primarily from insufficient descriptions of the algorithms employed and data processing [14, 16, 17, 22, 23]. Regarding the reference standard, 12 studies were rated as a high or unclear risk of bias due to the absence of a well-defined or appropriate reference method for validating the automated assessments [14–17, 19, 20, 22–27]. Finally, in the flow and timing domain, 11 studies were rated with a high or unclear risk of bias because they omitted reporting the uniform reference standard and reproducibility analyses [15–26]. A detailed risk of bias of the included studies with reasons is summarized in Supplementary Table S5.

**Table 1:** Bias and applicability assessment of included studies using tailored QUADAS-2 tool.

No.	Author/year	Risk of bias				Applicability concerns		
		Patient selection	Index test	Reference standard	Flow and timing	Patient selection	Index test	Reference standard
1	Darvann *et al*./2011 [14]	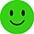	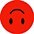	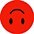	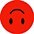	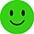	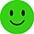	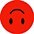
2	Verhoeven *et al*./2013 [15]	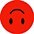	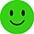	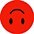	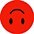	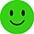	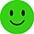	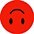
3	Alqattan *et al*./2015 [16]	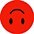	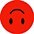	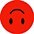	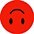	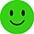	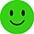	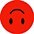
4	Patel *et al*./2015 [17]	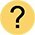	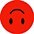	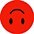	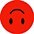	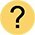	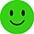	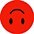
5	Sukno *et al*./2015 [18]	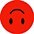	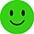	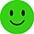	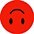	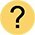	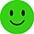	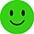
6	Liang *et al*./2017 [19]	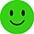	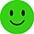	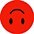	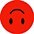	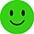	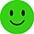	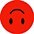
7	Al-Rudainy *et al*./2018 [20]	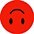	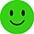	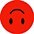	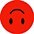	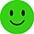	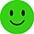	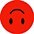
8	Ekrami *et al*./2018 [21]	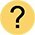	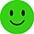	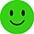	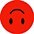	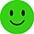	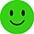	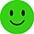
9	Lin *et al*./2019 [22]	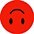	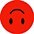	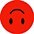	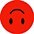	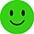	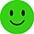	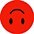
10	Bernini *et al*./2020 [23]	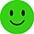	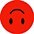	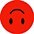	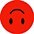	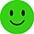	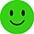	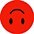
11	Hallac *et al*./2020 [24]	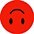	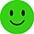	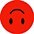	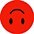	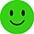	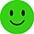	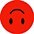
12	Gkantidis *et al*./2023 [25]	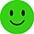	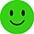	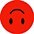	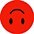	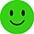	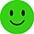	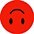
13	Zhao *et al*./2023 [26]	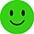	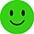	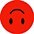	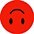	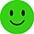	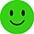	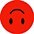
14	Yang *et al*./2025 [27]	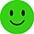	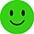	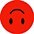	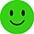	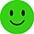	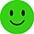	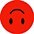

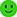
, risk/concern; 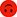, high risk/concern; 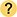, unclear risk/concern.

### Level of evidence

According to the GRADE approach, the overall certainty of evidence was rated as very low (Table 2). This judgment was based on serious limitations across several domains, including a high risk of bias, significant inconsistency (likely reflecting methodological heterogeneity), and serious imprecision (partly attributable to the lack of validity or reliability validation in multiple studies).

**Table 2:** GRADE analysis.

Certainty of assessment for clinical applicability							Impact	Certainty
No. of studies	Study design	Risk of bias	Inconsistency	Indirectness	Imprecision	Other considerations		
14	Cross-sectional study	Serious^a^	Serious^b^	Not serious	Serious^c^	None	Template-based methods are recommended for 3D facial asymmetry assessment due to their mathematically robust registration that mitigates the “Pinocchio effect” across severity levels. For mild asymmetry n clinical settings with limited technical expertise, commercial software-supported original–mirror alignment offers a practical alternative.	**⊕**○○○ Very low

^a^Most of the studies lacked a validated reference standard to prove the automated method's accuracy or the reliability metrics undermining reproducibility.

^b^Methodological heterogeneity may exist due to differences in 3D imaging techniques and algorithms for assessing facial asymmetry.

^c^Eight studies had questionable accuracy due to the lack of accuracy validation, and five had questionable internal validity due to the lack of the reliability assessment.

### Study characteristics

The demographic and methodological characteristics of the included studies are summarized in Tables 3 and 4, with further details provided in Supplementary Tables S6 and S7. This set of studies spanned a 14-year publication period from 2011 to 2025, with all articles published in English. The studies were predominantly cross-sectional in design and universally focused on developing or validating novel methods for the automated quantification of facial asymmetry. Stereophotogrammetry served as the principal imaging modality (*n* = 11 studies) [14, 15, 17, 19-26], including one study utilizing dynamic stereophotogrammetry [24]. Additional modalities included laser scanning (*n* = 2) and structured light (*n* = 1) [16, 18, 27]. Preprocessing to remove non-facial structures (e.g. ears, hair) was performed manually in most studies (*n* = 7) [15, 17, 20, 22, 23, 25, 26], while two studies accomplished this automatically via algorithm [19, 21]. Facial asymmetry analysis primarily relied on commercial software solutions (*n* = 9) [15–17, 20, 23-27], though some studies also employed custom algorithms (*n* = 5) [14, 18, 19, 21, 22]. While most studies reported fundamental sample characteristics, such as size, gender distribution, and age range, details regarding methodological rigor were often insufficient. Only two studies reported performing sample size calculations [26, 27], and three stated randomization was used for participant selection without detailing the actual methodology [25-27].

**Table 3:** Summary demographic characteristics of the included studies.

No.	Author/year	Study design	Study objective	Study population	Sample size calculation	Sample size	Sampling method
1	Darvann *et al*./2011 [14]	Cross-sectional	Develop an automated facial asymmetry quantification method for JIA patients	JIA patients with unilateral TMJ involvement and Healthy children	NR	44	Patients: consecutive Healthy children: NR
2	Verhoeven *et al*./2013 [15]	NR	Introduce and validate an automated facial asymmetry quantification method for post-mandibular reconstruction patients	Patients who underwent mandibular reconstruction and healthy volunteers	NR	39	NR
3	Alqattan *et al*./2015 [16]	Cross-sectional	Establish adult facial asymmetry reference ranges using two methods and compare their diagnostic performance clinically	Orthodontic patients with marked facial asymmetry and students/staff of Cardiff Dental Hospital	NR	88	NR
4	Patel *et al*./2015 [17]	Retrospective	Develop an automated facial asymmetry quantification method	Orthodontic patients	NR	58	NR
5	Sukno *et al*./2015 [18]	NR	Introduce and validate an automated facial asymmetry quantification method	Healthy volunteers	NR	100	NR
6	Liang *et al*./2017 [19]	NR	Develop and validate automated methods to quantify nasal asymmetry and nasal normalcy for UCL patients	UCL patients and control group from Seattle Children’s Hospital and normal reference from 3D Facial Norms Database	NR	63	UCL patients: consecutive Control group and normal reference: NR
7	Al-Rudainy *et al*./2018 [20]	NR	Evaluate regional facial asymmetry changes in UCLP patients before and after surgery	UCLP infants from the Royal Hospital for Sick Children, Edinburgh, UK	NR	26	NR
8	Ekrami *et al*./2018 [21]	Cross-sectional	Develop an automated facial asymmetry quantification method	Penn State University dataset	NR	430	NR
9	Lin *et al*./2019 [22]	NR	Develop a transfer learning-based system to quantify facial symmetry from 3D contour maps	Patients who underwent orthognathic surgery from Chang Gung Memorial Hospital in Taiwan and normal population for training/validation/test dataset	NR	195	NR
10	Bernini *et al*./2020 [23]	Retrospective	Investigate associations between facial asymmetry and asymmetrical TMJ osseous destruction	JIA patients from a university clinic and children’s hospital	NR	76	Consecutive
11	Hallac *et al*./2020 [24]	Cross-sectional	Dynamic analysis of facial asymmetry in healthy pediatric populations	Healthy pediatric volunteers	NR	36	NR
12	Gkantidis *et al*./2023 [25]	Prospective study	Validate an automated method for facial asymmetry assessment and MSP definition	Orthodontic patients from the University of Bern, Switzerland	NR	20	Random
13	Zhao *et al*./2023 [26]	NR	Quantify hard-soft tissue symmetry across menton deviations and sagittal skeletal patterns	Patients from the Stomatology Hospital of Xian Jiaotong University, China	Yes	270	Random
14	Yang *et al*./2025 [27]	Cross-sectional	Validate the accuracy of an automated facial asymmetry quantification method	Orthodontic patients from Peking University Stomatological Hospital	Yes	24	Random

JIA, juvenile idiopathic arthritis; TMJ, temporomandibular joint; NR, not reported; UCL, unilateral cleft lip; UCLP, unilateral cleft lip and palate.

**Table 4:** Summary methodological characteristics of the included studies.

No.	Author/year	Imaging modality	Software platform	Image preprocessing	Alignment approach	Core algorithm	Initial landmarking	Asymmetry metrics	Reference standard	Reliability assessment
1	Darvann *et al*./2011 [14]	SP	CA	NR	OM alignment and template-based	B-spline-based non-rigid registration	NR	MAD	vs landmark-based method	NR
2	Verhoeven *et al*./2013 [15]	SP	CS	Manual	OM alignment	ICP algorithm	Manual	MAD	NR	Rep + 2Obs
3	Alqattan *et al*./2015 [16]	Laser scanning	CS	NR	OM alignment	ICP algorithm	NR	MAD + %≤0.5 mm	NR	Rep
4	Patel *et al*./2015 [17]	SP	CS	Manual	OM alignment	LM algorithm	Manual	RMS	NR	Rep
5	Sukno *et al*./2015 [18]	Laser scanning	CA	NR	Template-based	Template-mapping + HM-DW-LMedS algorithm	NR	MAD	vs synthetic asymmetry patterns	NR
6	Liang *et al*./2017 [19]	SP	CS + CA	Auto	Depth-stratified	NR	N/A	BAD in different depth	vs experts	NR
7	Al-Rudainy *et al*./2018 [20]	SP	CS	Manual	OM alignment	ICP algorithm	Manual	90% of MAD	NR	Rep
8	Ekrami *et al*./2018 [21]	SP	CA	Auto	Template-based	Template-mapping and robust PA	Manual	MSD	vs true simulated values	NR
9	Lin *et al*./2019 [22]	SP	CA	Manual	Depth-stratified	CNN	N/A	Overlap feature of bilateral contour line	vs diverse reviewers	NR
10	Bernini *et al*./2020 [23]	SP	CS	Manual	OM alignment	ICP algorithm	Semi-auto	HD for chin area	NR	Rep
11	Hallac *et al*./2020 [24]	4D SP	CS	NR	Template-based	Template-mapping	Manual	Bilateral difference of semi-landmarks displacement	NR	2Obs
12	Gkantidis *et al*./2023 [25]	SP	CS	Manual	OM alignment	ICP algorithm	NR	MAD for chin area	NR	Rep
13	Zhao *et al*./2023 [26]	SP	CS	Manual	OM alignment	ICP algorithm	NR	RMS	NR	Rep + 2Obs
14	Yang *et al*./2025 [27]	Structured light scanning	CS + open-source tool	N/A	Landmark-based	Template-mapping + robust PA	N/A	|Angle_R_−Angle_L_|/Angle_L_ × 100%	vs OM alignment method	Rep

SP, stereophotogrammetry; CA, custom algorithms; OM, original-mirror; MSP, mid-saggital plane; NR, not reported; ICP, Iterative Closest Point; MAD, mean absolute distance; CS, commercial software; 2Obs, two observers; Rep, repetition; LM, Levenberg-Marquardt; %≤0.5 mm, the percentage of surface points within 0.5 mm; RMS, root mean square; N/A, not applicable; HM-DW-LMedS, Hemispheres-Distance-weighted Least Median of Squares; auto, automated; BAD, bilateral area difference; PA, procrustes analysis; MSD, mean signed distance; *r*^2^, coefficient of determination; CNN, convolutional neural network; HD, Hausdorff distance.

### Outcome synthesis

Outcomes are synthesized in the following section, organized based on methodology, validity, and reliability.

#### Methodologies used for assessing facial asymmetry

The included studies utilized various computational methodologies for assessing facial asymmetry, which can be categorized into four primary approaches: landmark-based (*n* = 1) [27], depth-stratified (*n* = 2) [19, 22], original-mirror alignment (*n* = 8) [14-17, 20, 23, 25, 26], and template-based (*n* = 4) methods [14, 18, 21, 24].

Landmark-based methods quantify asymmetry through anatomical landmarks placed on critical facial features. Yang *et al*. [27] exemplified this approach by automatically transferring 34 predefined landmarks from a template to patient scans using MeshMonk toolbox. The interconnected landmarks created various angles, allowing for the computation of an Angle Asymmetry Index by comparing bilateral 3D angular differences.

Depth-stratified methods analyze serial cross-sections of the 3D facial surface in the coronal plane, quantifying asymmetry by comparing bilateral geometric features. Liang *et al*. [19] employed this approach by automatically detecting 83 landmarks to define a MSP, cropping the nasolabial region, and slicing it into 14 coronal planes. They calculated a depth area difference metric by summing the absolute area differences between the left and right nasal regions across all planes. Similarly, Lin *et al*. [22] extracted full-face contour maps at uniform intervals, ensuring consistent height and spacing. After dividing the contour maps into halves along the MSP, the researchers used bilateral contour maps differences as both symmetry indicators and input features for training a Convolutional Neural Network to automate symmetry assessment.

Original-mirror alignment methods create a mirrored surface of the original facial scan and align it with the original surface, based on a dense set of semi-landmarks. The core process involves precise registration, typically employing rigid iterative closest point (ICP) algorithms or non-rigid surface registration techniques. Asymmetry is quantified by analyzing the positional differences between the original surface and its aligned mirrored image. Rigid ICP registration iteratively establishes correspondence between the original and mirrored surfaces within a defined tolerance threshold, requiring initial alignment using the stable reference areas or anatomical landmarks due to sensitivity to initial positioning. Implementation strategies for initial alignment varied across studies: Verhoeven *et al*. [15] and Patel *et al*. [17] utilized forehead, nasal dorsum, and zygoma, while Al-Rudainy *et al*. [20] employed nine facial landmarks. Global ICP registration processed entire facial surfaces in most studies [15, 16, 20, 25, 26], though Bernini *et al*. [23] used five semi-automatic landmarks to achieve initial alignment specifically within the midfacial region before performing a regional ICP registration of this area. Non-rigid techniques, such as B-spline-based registration in the study of Darvann *et al*. [14], permitted elastic deformation for complex anatomical correspondences, quantifying asymmetry as the distance between original and mirrored surface. Besides, Patel *et al*. [17] implemented the Levenberg-Marquardt algorithm to achieve registration.

Template-based methods mainly rely on mapping templates with a dense set of known semi-landmarks onto the target surfaces through the registration of these templates and the target surfaces, thereby transferring the knowledge of left and right correspondence in the template to the individuals.

Darvann *et al*. [14] used B-spline non-rigid registration to minimize distances between a symmetric atlas and patient-specific symmetric surfaces constructed from original-mirror averages, quantifying asymmetry as the inter-surface distance. After template mapping, Sukno *et al*. [18] aligned original and mirrored faces using a Hemispheres-Distance-weighted Least Median of Squares (HM-DW-LMedS) algorithm, which strategically assigned higher weights to semi-landmarks near the facial MSP and lower weights to peripheral regions.

The procrustes analysis (PA) algorithm aligns original and mirrored facial surfaces via rotation, translation, and scaling to minimize the distances between the predefined landmarks on both surfaces [28]. Ekrami *et al*. [21] created a symmetric anthropometric mask with 7160 semi-landmarks based on an averaged facial form of 400 Western Australian individuals. They mapped the symmetric mask onto target scans using rigid ICP and non-rigid deformation. After mirroring the scan by reversing the sign of the x-coordinate of each point on the original face, a robust PA algorithm was employed. This algorithm assigned higher weights to semi-landmarks in the symmetry regions and lower weights to those in the asymmetry regions. Directional asymmetry (DA) was calculated as the mean signed distance (MSD) between average surface of the population and average surface of original and mirrored one, and fluctuating asymmetry (FA) was derived by subtracting DA from MSD between original and mirrored surface. Hallac *et al*. [24] registered an 884-landmark template to resting-position scans using PA based on 32 landmarks, then tracked bilateral displacement of semi-landmark from rest to maximal smile using dynamic stereophotogrammetry to assess bilateral asymmetry.

#### The validity and reliability of automated evaluation for facial asymmetry

Six studies evaluated the validity of automated asymmetry quantification through benchmarking against established standards [14, 18, 19, 21, 22, 27]. Comparative analyses demonstrated strong correspondence with alternative methodologies. Darvann *et al*. [14] reported a correlation coefficient (*r*) of 0.92 between automated original-mirror alignment and manual landmark-based methods, while Yang *et al*. [27] demonstrated classification consistency (87.5%–100%) across facial regions between automated landmark-based and original-mirror alignment methods. By validation against synthetic references, Ekrami *et al*. [21] reported a coefficient of determination (*r*^2^) of 0.99, and Sukno *et al*. [18] reported a *r* >0.9 between the template-based results and the simulated ground truth. Clinical validation included Liang *et al*.'s [19] depth-stratified metrics showing *r* = 0.70 correlation with clinician-ranked cleft severity, and Lin *et al*.'s [22] 78.85% classification accuracy against human raters with 98.63% agreement within clinical tolerance thresholds for their depth-stratified approach.

Methodological reliability was examined across nine studies employing repeated measurements and multi-observer designs [15-17, 20, 23-27]. Minimal measurement variation was consistently observed—Verhoeven *et al*. [15] documented intra-observer differences of 0.02 mm and inter-observer differences of 0.04 mm, while Alqattan *et al*. [16] achieved sub-micrometer precision (<0.005 mm) in repeated tests. Regional analyses showed comparable stability, with mean absolute differences <0.01 mm in chin asymmetry quantification and non-significant variations across repetitions (*P* > .05) [20, 25]. Statistical agreement metrics included the intraclass correlation coefficient (ICC) values ranging from 0.81 to 0.95 [23, 26], Lin’s concordance correlation coefficient (CCC) of 0.98 for dynamic assessments [24], and *κ* statistics between 0.664 and 1.00 [27].

## Discussion

This systematic review presents the first comprehensive synthesis of methodologies for automated assessment of facial asymmetry using 3D static or dynamic facial scans. Our analysis reveals a diverse landscape of approaches, each exhibiting distinct strengths and limitations that warrant careful consideration in clinical and research contexts.

### Different types of images and asymmetry

The transition from 2D photography to 3D imaging has significantly advanced asymmetry evaluation, overcoming inherent 2D limitations including anatomical superimposition, magnification errors, and perspective distortion [29, 30]. Contemporary 3D acquisition systems, categorized by their operating principles into laser-based scanning, stereophotogrammetry, structured-light scanning, and RGB-D sensors, provide anatomically faithful representations of facial morphology [31]. Stereophotogrammetry predominates in facial asymmetry research due to its metrological precision and reliability in capturing complex facial surface geometry [14, 15, 17, 19-26, 31, 32].

A critical distinction exists between static and dynamic 3D acquisition. While static scans capture resting morphology, dynamic systems record facial motion—a clinically significant dimension that remains underexplored. Dynamic assessment is particularly crucial for detecting subtle abnormalities in patients with facial palsy or stroke [33], and for evaluating post-surgical scarring in cleft patients, where restricted muscle movement may exacerbate asymmetry [34]. Moreover, even in normative populations, asymmetries often become more perceptible during facial expression than at rest. Despite this, only one included study utilized dynamic stereophotogrammetry for smile kinematics analysis [24]. Thus, automated dynamic asymmetry quantification represents an essential frontier for pathology detection and treatment planning, yet current methodologies remain underdeveloped compared with static approaches.

From a morphological perspective, facial asymmetry is classified into three distinct types: DA, FA, and anti-symmetry (AS) [21, 35]. DA is defined by a population-level, systematic bias favoring one side, often due to genetic or adaptive factors. AS is also considered to be population-level traits, but its direction (left or right) varies randomly. In contrast, FA constitutes small, random deviations from perfect bilateral symmetry or the population's DA, typically resulting from developmental instability or environmental stressors during growth.

Clinically, DA is the primary focus of orthodontic and orthognathic treatment. Pronounced conditions such as hemifacial microsomia, unilateral condylar hyperplasia, and significant mandibular deviations are manifestations of DA that compromise function, aesthetics, and patient well-being, thus necessitating corrective intervention. Consequently, most studies included in this review focused on DA assessment. In contrast, minor FA is considered a normal component of biological variation and often falls within an acceptable aesthetic range, though elevated levels may signal underlying developmental issues [36, 37]. Due to its subtlety and difficulty of accurate quantification, only one included study specifically addressed FA assessment [21].

### Clinical applicability of automated facial asymmetry assessment methods

Landmark-based methods demonstrate significant clinical utility through automated landmark placement, which eliminates manual landmarking errors and substantially reduces processing time. The approach exemplified by Yang *et al*. offers advantages through its independence from MSP definition, a process lacking standardized methodology, thereby avoiding associated errors [27, 38]. Its conceptual simplicity and minimal preprocessing requirements, which are limited to automated landmark placement, further enhance its accessibility. However, fundamental limitations remain, as the analysis is restricted to landmark-adjacent regions, neglecting complex anatomical zones like the nasal structure that cannot be adequately characterized by sparse points. Current implementations often rely on template-based landmark transfer requiring manual intervention for initial alignment [39].

Depth-stratified methods utilize serial coronal cross-sections of the 3D facial surface, which contain more information on the dense face meshes, compared with the landmark-based method. Liang *et al*. [19] demonstrated this capability through automated landmark detection for MSP construction and region-of-interest (ROI) cropping. Lin *et al*. [22] further advanced the paradigm as the only reviewed study employing deep learning for asymmetry classification. However, critical preprocessing requirements present challenges: the removal of non-facial structures and the standardization of facial scan orientations remain manual in many studies. The methodology's inherent dependence on MSP definition, frequently determined by a limited number of landmarks, risks inaccuracies due to deviations from the true MSP and errors in landmark identification [38]. Furthermore, although discrete contour sampling captures more data than landmark-based methods, it may overlook subtle morphological variations between cross-sections.

Original-mirror alignment methods facilitate a comprehensive facial analysis by allowing for an extensive comparison of point clouds. ROI segmentation, such as the nasolabial complex and chin, enables precise quantification of regional asymmetry [15, 16, 20, 23, 25, 26]. This specificity enhances diagnostic accuracy and improves communication with patients, particularly concerning asymmetries that extend beyond the scope of orthodontics (e.g. periorbital regions) [16]. Regarding clinical feasibility, results showed that many commercial software programs, such as Geomagic Wrap, Rapidform, and 3D Slicer, incorporate in-house algorithms to analyze facial asymmetry, making them easier for clinicians to adopt [15-17, 20, 23, 25, 26].

However, the preprocessing for global registration necessitates manual removal of non-facial structures, and the preprocessing for regional registration necessitates manual ROI extraction [15, 17, 20, 23, 25, 26]. Algorithmically, the predominant technique ICP registration exhibits critical sensitivity to initial alignment. Poor initialization risks convergence to local minima during optimization, yielding suboptimal solutions that necessitate manual landmarking or ROI segmentation [40]. More fundamentally, ICP registration relies on nearest-neighbor correspondence rather than anatomical homology. While sufficient for minor asymmetries, this approach fails catastrophically in cases of severe asymmetry due to the “Pinocchio effect,” where pronounced localized deformations (e.g. hemifacial microsomia) dominate least-squares minimization [41-44]. Just as a protruding nose distorts the registration between honest and lying Pinocchio faces, causing an underestimation of nasal differences while exaggerating disparities elsewhere, significant craniofacial anomalies exert a similarly disproportionate influence on superimposition. Consequently, as Hajeer *et al*. [45] cautioned, severe preoperative asymmetries can distort the best-fit alignment produced by the ICP algorithm, potentially leading to misleading postoperative “improvements” in untreated regions when comparing pre- and post-interventional states.

Template-based methods automate anatomically meaningful correspondence through symmetric template mapping, simultaneously addressing non-facial structure removal [21]. Both Sukno *et al*.'s [18] HM-DW-LMedS and Ekrami *et al*.'s [21] robust PA employ weighted registration frameworks that strategically assign higher weights to landmarks in symmetry-critical regions (near MSP or symmetric zones) and lower weights to peripheral/asymmetric areas. These weighted registration frameworks demonstrate significant potential for mitigating the “Pinocchio effect” in severe craniofacial deformities by preventing localized anomalies from dominating the alignment process.

Nevertheless, critical limitations hinder clinical translation. The initial template-mapping registration universally requires manual landmarking for initial alignment [21, 24]. Furthermore, inherent mapping accuracy thresholds (e.g. 1.3 mm in Ekrami *et al*.) establish detection limits, below which asymmetries become indistinguishable from algorithmic noise [21]. Critically, the 3D template constructed by a single ethnicity, such as Ekrami's template derived exclusively from 400 Western Australian individuals, limits cross-population generalizability [21]. Given documented inter-ethnic facial variations [46], applying such templates to diverse populations (e.g. African or Asian) introduces risks of mapping inaccuracies, underscoring the need for ethnic-specific template development. Additionally, the registration using the template- based method in many current studies necessitates a basic knowledge of coding, making it less accessible for clinicians [14, 18, 21].

Key characteristics of automated assessment methodologies are summarized in Table 5. Clinical recommendations prioritize original-mirror and template-based techniques, as they utilize dense semi-landmarks for comprehensive analysis, unlike the sparse data in landmark-based or depth-stratified approaches. Template-based methods are preferred across the severity spectrum when technical resources permit, as anatomically informed correspondence and weighting strategies reduce susceptibility to localized deformities during registration. It should be emphasized that the original–mirror workflow is mathematically valid; limitations arise from its common implementations that rely on unconstrained ICP-based best-fit registration, whose nearest-neighbor correspondence and unweighted least-squares objective can be vulnerable to localized extreme deformities. Consequently, commercial software-supported original–mirror alignment may only be considered for mild asymmetry, where deviations are small and are less likely to skew the registration objective, rendering nearest-neighbor best-fit alignment (e.g. ICP) approximately valid.

**Table 5:** Comparative analysis of automated assessment methodologies for 3D facial asymmetry.

Assessment methods	Data foundation	Advantages	Limitations
Landmark-based method	Sparse and limited anatomical landmarks	Conceptually simple Minimal preprocessing requirements	Analysis restricted to landmark-adjacent regions Inadequate for complex anatomical surfaces
Depth-stratified method	Serial coronal cross-sections of the 3D facial surface	Utilizes more information beyond sparse points Potential for deep learning automation	Dependent on accurate MSP definition Manual preprocessing (non-facial structure removal, orientation standardization) Potential loss of inter-slice morphological data
Original-mirror alignment method	Dense semi-landmarks	Utilizes dense surface data beyond sparse points and cross-sections High clinical feasibility via commercial software	Manual preprocessing (non-facial structure removal, initial alignment, and ROI extraction) “Pinocchio effect” causes significant errors in severe asymmetry cases
Template-based method	Dense semi-landmarks with anatomical correspondence	Utilizes dense surface data beyond sparse points and cross-sections Automated anatomically meaningful correspondence Mitigates “Pinocchio effect” via weighted registration Suitable for severe asymmetries	Requires manual landmarking for initial template mapping Demographic bias in templates limits cross-population generalizability Requires programming proficiency for implementation

Importantly, a clear distinction exists for severe cases. In severe cases, localized extreme deformities can disproportionately influence least-squares optimization (the “Pinocchio effect”), which distorts global alignment and generates spurious deviations in otherwise symmetric regions. While this may provide a qualitative indication of the affected area, it is unsuitable for precise quantitative assessment or longitudinal comparison due to the systematic registration bias. Therefore, unconstrained ICP should be avoided for severe deformity in favor of the more robust template-based methods.

Nevertheless, these recommendations must be interpreted with caution, as the supporting evidence was rated as very low certainty under the GRADE framework (Table 2). Generalizability is constrained by significant heterogeneity in imaging and algorithms, while QUADAS-2 assessment revealed high risks of bias due to the absence of reliability testing. Additionally, the lack of a validated threshold for the “Pinocchio effect” necessitates clinical judgment. Consequently, these findings do not establish definitive standards but serve as preliminary guidance to prioritize future research and clinical application.

### Validity and reliability of automated assessment methods

Regarding validity, while several studies report strong performance, these findings warrant cautious interpretation. In the absence of a universal gold standard, the validity reflect validation against study-specific reference frameworks. Thus, our analysis prioritizes the methodological strengths, limitations, and implementation assumptions of each approach and support future standardization and validation efforts. The validity metrics is intrinsically linked to the selection of ground truth. Synthetically generated asymmetry patterns [e.g. Ekrami *et al*. (*r*^2^ = 0.99 for FA); Sukno *et al*. (*r* > 0.9 across 25 patterns)] enable precise algorithmic validation against a known deformation field, but may not fully capture the biological variability observed in real-world scenarios [18, 21]. Conversely, human-referenced standards—such as expert-ranked cleft severity and average ratings from 50 diverse reviewers [Liang *et al*. (*r* = 0.70); Lin *et al*. (78.85% accuracy)]—reveal moderate-to-strong correlations, suggesting automated methods can approximate but not yet fully replicate clinical judgment [19, 22].

Concerning reliability, the literature demonstrates generally good agreement metrics for repeated measurements and inter-observer assessments [15-17, 20, 23-27]. Nevertheless, critical workflow elements threaten result reproducibility: manual non-facial structure removal for image preprocessing and landmark identification for initial alignment and region analysis inherently introduce operator-dependent subjectivity [14-17, 20-26]. This methodological vulnerability is compounded by the “Pinocchio effect” in severe asymmetries, where local deformations disproportionately influence registration outcomes and thereby impact measurement consistency across evaluations. Consequently, while reported ICC (e.g. 0.81–0.95 in Bernini *et al*. [23]), CCC (0.98 in Hallac *et al*. [24]), and *κ* values (0.664–1.00 in Yang *et al*. [27]) indicate technical precision, their real-world generalizability remains constrained by these persistent human-intervention requirements. Given the absence of a universal gold standard, reliability and reproducibility metrics provide a practical basis for assessing methodological robustness and should be prioritized and standardized in future validation studies.

### Limitations and future directions

The primary limitation of this review is the high risk of bias within the included studies. The QUADAS-2 assessment flagged 8 of 14 studies as high risk, largely due to the absence of validity or reliability evaluations. This results in a “very low” certainty of evidence under the GRADE framework; consequently, these recommendations must be viewed as preliminary guidance.

Beyond study quality, contemporary methodologies face critical technical constraints. First, most methods rely on static morphology, overlooking diagnostic dynamic asymmetry in conditions like facial palsy. Second, workflow dependencies persist through manual requirements for alignment, MSP construction, and segmentation, which introduce subjectivity. Third, demographic biases exist in template-based methods where data from single-ethnicity fail to represent diverse ethnic groups. Finally, a critical evidence gap also remains regarding the “Pinocchio effect,” as no quantitative threshold currently exists to determine when asymmetry becomes severe enough to cause ICP failure.

Future research must address these gaps by developing integrated deep learning pipelines that automate the complete workflow from preprocessing to quantification to minimize operator dependence. To improve precision, MSP definitions should evolve from sparse landmarks to dense semi-landmark constellations utilizing weighted PA algorithms [47]. Validation frameworks must also be standardized by adopting multi-modal approaches that combine synthetic datasets with expert evaluations and rigorous reliability reporting. Furthermore, the field must prioritize clinical translation by shifting from opaque, commercial “black-box” workflows to transparent, open-source tools. Ultimately, pairing these accessible technologies with standardized validation is essential to ensure trustworthy adoption in routine practice.

## Conclusion

Given the “very low” certainty of evidence identified by the GRADE framework, these recommendations should be interpreted as preliminary and based primarily on comparative technical reasoning. For clinicians, method selection should balance both mathematical robustness and clinical practicality. Template-based, correspondence-constrained approaches with weighted registration appear preferable across the severity spectrum when resources allow, as they reduce susceptibility to the “Pinocchio effect” and are more likely to remain stable in severe deformity. Conversely, the original–mirror workflow relies on unconstrained best-fit/ICP registration, which is prone to systematic errors in severe asymmetry. Future research should prioritize dynamic analysis, fully automated workflows, and multi-modal validation. Crucially, bridging the gap between algorithmic innovation and transparent, user-friendly clinical tools is essential to transition automated facial asymmetry assessment from research to routine practice, thereby enhancing diagnostic precision in orthodontics and maxillofacial surgery.

## Supplementary Material

cjag012_Supplementary_Data

## Data Availability

The data underlying this article will be shared on reasonable request to the corresponding author.

## References

[1] Baudouin JY, Tiberghien G. Symmetry, averageness, and feature size in the facial attractiveness of women. Acta Psychol 2004;117:313–32. 10.1016/j.actpsy.2004.07.002 15500809

[2] Tam TKM, Guo R, Liu H, et al Hard and soft tissue asymmetry in patients with skeletal class III malocclusion: a cone-beam computed tomography study. Diagnostics 2023;13:869. 10.3390/diagnostics13050869 36900013 PMC10000951

[3] Burstone CJ . The integumental profile. Am J Orthod 1958;44:1–25. 10.1016/S0002-9416(58)90178-7

[4] Nanda RS, Ghosh J. Facial soft tissue harmony and growthin orthodontic treatment. Semin Orthod 1995;1:67–81. 10.1016/S1073-8746(95)80094-8 8935046

[5] Wan SY, Tsai PY, Lo LJ. Quantifying perceived facial asymmetry to enhance physician-patient communications. Appl Sci 2021;11:8398. 10.3390/app11188398

[6] Lee ST, Mori Y, Minami K et al Does skeletal surgery for asymmetric mandibular prognathism influence the soft tissue contour and thickness? J Oral Maxillofac Surg 2013;71:1577–87. 10.1016/j.joms.2013.04.008 23800674

[7] Wang Y, Wu W, Christelle M, et al Automated localization of mandibular landmarks in the construction of mandibular median sagittal plane. Eur J Med Res 2024;29:84. 10.1186/s40001-024-01681-2 38287445 PMC10823719

[8] Lahoud P, EzEldeen M, Beznik T et al Artificial intelligence for fast and accurate 3-dimensional tooth segmentation on cone-beam computed tomography. J Endod 2021;47:827–35. 10.1016/j.joen.2020.12.020 33434565

[9] Qu W, Qiu Z, Lam KC, et al Artificial intelligence-assisted identification and assessment of mandibular asymmetry on panoramic radiography. Am J Orthod Dentofac Orthop 2025;168:14–23. 10.1016/j.ajodo.2025.01.018 40047777

[10] Kazimierczak N, Kazimierczak W, Serafin Z et al Skeletal facial asymmetry: reliability of manual and artificial intelligence-driven analysis. Dentomaxillofac Radiol 2024;53:52–9. 10.1093/dmfr/twad006 38214946 PMC11003660

[11] Page MJ, McKenzie JE, Bossuyt PM et al The prisma 2020 statement: an updated guideline for reporting systematic reviews. BMJ 2021;372:n71. 10.1136/bmj.n71 33782057 PMC8005924

[12] Whiting PF, Rutjes AWS, Westwood ME et al Quadas-2: a revised tool for the quality assessment of diagnostic accuracy studies. Ann Intern Med 2011;155:529–U104. 10.7326/0003-4819-155-8-201110180-00009 22007046

[13] Ryan R, Hill S. How to grade the quality of the evidence. Cochrane Consumers and Communication Group. http://cccrg.cochrane.org/author-resources (28 October, 2025, date last accessed).

[14] Darvann TA, Hermann NV, Demant S, et al Automated quantification and analysis of facial asymmetry in children with arthritis in the temporomandibular joint. In: Presented at: 2011 8th IEEE International Symposium on Biomedical Imaging: From Nano to Macro; Chicago, IL: IEEE; 2011. 10.1109/ISBI.2011.5872615

[15] Verhoeven TJ, Coppen C, Barkhuysen R et al Three dimensional evaluation of facial asymmetry after mandibular reconstruction: validation of a new method using stereophotogrammetry. Int J Oral Maxillofac Surg 2013;42:19–25. 10.1016/j.ijom.2012.05.036 22939875

[16] Alqattan M, Djordjevic J, Zhurov AI et al Comparison between landmark and surface-based three-dimensional analyses of facial asymmetry in adults. Eur J Orthod 2015;37:1–12. 10.1093/ejo/cjt075 24152377

[17] Patel A, Islam SMS, Murray K et al Facial asymmetry assessment in adults using three-dimensional surface imaging. Prog Orthod 2015;16:36. 10.1186/s40510-015-0106-9 26490376 PMC4614853

[18] Sukno FM, Rojas MA, Waddington JL, et al On the quantitative analysis of craniofacial asymmetry in 3D. In: Presented at: 2015 11th IEEE International Conference and Workshops on Automatic Face and Gesture Recognition (FG), Vol. 1; Ljubljana, Slovenia: IEEE; 2015. 10.1109/FG.2015.7163143

[19] Liang S, Shapiro L, Tse R. Measuring symmetry in children with cleft lip. Part 3: quantifying nasal symmetry and nasal normalcy before and after unilateral cleft lip repair. Cleft Palate Craniofac J 2017;54:602–11. 10.1597/16-035 27580380

[20] Al-Rudainy D, Ju XY, Stanton S et al Assessment of regional asymmetry of the face before and after surgical correction of unilateral cleft lip. J Craniomaxillofac Surg 2018;46:974–8. 10.1016/j.jcms.2018.03.023 29752048

[21] Ekrami O, Claes P, White JD et al Measuring asymmetry from high-density 3D surface scans: an application to human faces. PLoS One 2018;13:e0207895. 10.1371/journal.pone.0207895 30586353 PMC6306226

[22] Lin HH, Lo LJ, Chiang WC. A novel assessment technique for the degree of facial symmetry before and after orthognathic surgery based on three-dimensional contour features using deep learning algorithms. In: presented at: Proceedings of the 2019 9th International Conference on Biomedical Engineering and Technology (ICBET 2019); Tokyo, Japan: Association for Computing Machinery (ACM); 2019:170–3. 10.1145/3326172.3326222

[23] Bernini JM, Kellenberger CJ, Eichenberger M et al Quantitative analysis of facial asymmetry based on three-dimensional photography: a valuable indicator for asymmetrical temporomandibular joint affection in juvenile idiopathic arthritis patients? Pediatr Rheumatol Online J 2020;18:10. 10.1186/s12969-020-0401-y 32005249 PMC6995089

[24] Hallac RR, Thrikutam N, Chou PY et al Kinematic analysis of smiles in the healthy pediatric population using 3-dimensional motion capture. Cleft Palate Craniofac J 2020;57:430–7. 10.1177/1055665619887628 31726862

[25] Gkantidis N, Opacic J, Kanavakis G et al Facial asymmetry and midsagittal plane definition in 3D: a bias-free, automated method. PLoS One 2023;18:e0294528. 10.1371/journal.pone.0294528 38011159 PMC10681257

[26] Zhao JM, Xu YF, Wang JX et al 3-dimensional analysis of hard- and soft-tissue symmetry in a Chinese population. BMC Oral Health 2023;23:432. 10.1186/s12903-023-03163-z 37386472 PMC10308641

[27] Yang GC, Lyu L, Wen AN et al Comparison of mirroring and overlapping analysis and three-dimensional soft tissue spatial angle wireframe template in evaluating facial asymmetry. Bioengineering 2025;12:79. 10.3390/bioengineering12010079 39851353 PMC11761234

[28] Klingenberg CP, Barluenga M, Meyer A. Shape analysis of symmetric structures: quantifying variation among individuals and asymmetry. Evolution 2002;56:1909–20. 10.1111/j.0014-3820.2002.tb00117.x 12449478

[29] Nike E, Radzins O, Pirttiniemi P et al Evaluation of facial soft tissue asymmetric changes in class III patients after orthognathic surgery using three-dimensional stereophotogrammetry. Int J Oral Maxillofac Surg 2023;52:361–70. 10.1016/j.ijom.2022.06.022 35871879

[30] Pedersoli L, Dalessandri D, Tonni I et al Facial asymmetry detected with 3D methods in orthodontics: a systematic review. Open Dent J 2022;16:e187421062111251. 10.2174/18742106-v16-e2111251

[31] Petrides G, Clark JR, Low H et al Three-dimensional scanners for soft-tissue facial assessment in clinical practice. J Plast Reconstr Aesthet Surg 2021;74:605–14. 10.1016/j.bjps.2020.08.050 33082078

[32] D'Ettorre G, Farronato M, Candida E et al A comparison between stereophotogrammetry and smartphone structured light technology for three-dimensional face scanning. Angle Orthod 2022;92:358–63. 10.2319/040921-290.1 35015071 PMC9020391

[33] Alagha MA, Ayoub A, Morley S et al Objective grading facial paralysis severity using a dynamic 3D stereo photogrammetry imaging system. Opt Lasers Eng 2022;150:106876. 10.1016/j.optlaseng.2021.106876

[34] Gattani S, Ju XY, Gillgrass T et al An innovative assessment of the dynamics of facial movements in surgically managed unilateral cleft lip and palate using 4D imaging. Cleft Palate Craniofac J 2020;57:1125–33. 10.1177/1055665620924871 32419475 PMC7594373

[35] Klingenberg CP . Analyzing fluctuating asymmetry with geometric morphometrics: concepts, methods, and applications. Symmetry (Basel) 2015;7:843–934. 10.3390/sym7020843

[36] Klingenberg CP . Developmental Instability as a Research Tool: Using Patterns of Fluctuating Asymmetry to Infer the Developmental Origins of Morphological Integration. Developmental Instability: Causes and Consequences. Oxford: Oxford University Press, 2003. 10.1093/oso/9780195143454.003.0024

[37] Palme R, Strobeck C. Fluctuating Asymmetry Analyses Revisited. Developmental Instability: Causes and Consequences. Oxford: Oxford University Press, 2003. 10.1093/oso/9780195143454.003.0017

[38] Ajmera DH, Singh P, Leung YY et al Establishment of the mid-sagittal reference plane for three-dimensional assessment of facial asymmetry: a systematic review. Clin Oral Investig 2024;28:242. 10.1007/s00784-024-05620-7 PMC10995046 38575839

[39] Wen AN, Zhu YJ, Zheng SW et al Preliminary study on the method of automatically determining facial landmarks based on three-dimensional face template. Zhonghua Kou Qiang Yi Xue Za Zhi 2022;57:358–65. 10.3760/cma.j.cn112144-20210913-00409 35368162

[40] Wang Y, Solomon JM. Deep closest point: Learning representations for point cloud registration. In: Presented at: 2019 IEEE/CVF International Conference on Computer Vision (ICCV 2019); Seoul, South Korea: IEEE; 2019. 10.1109/ICCV.2019.00362

[41] Claes P, Walters M, Clement J. Improved facial outcome assessment using a 3D anthropometric mask. Int J Oral Maxillofac Surg 2012;41:324–30. 10.1016/j.ijom.2011.10.019 22103995

[42] Matthews HS, Burge JA, Verhelst PJR et al Pitfalls and promise of 3-dimensional image comparison for craniofacial surgical assessment. Plast Reconstr Surg Glob Open 2020;8:e2847. 10.1097/GOX.0000000000002847 33154878 PMC7605870

[43] Claes P, Daniels K, Walters M et al Dysmorphometrics: the modelling of morphological abnormalities. Theor Biol Med Model 2012;9:5. 10.1186/1742-4682-9-5 22309623 PMC3297492

[44] Claes P, Walters M, Vandermeulen D et al Spatially-dense 3D facial asymmetry assessment in both typical and disordered growth. J Anat 2011;219:444–55. 10.1111/j.1469-7580.2011.01411.x 21740426 PMC3187867

[45] Hajeer MY, Mao Z, Millett DT et al A new three-dimensional method of assessing facial volumetric changes after orthognathic treatment. Cleft Palate Craniofac J 2005;42:113–20. 10.1597/03-132.1 15748101

[46] Wen YF, Wong HM, Lin R et al Inter-ethnic/racial facial variations: a systematic review and Bayesian meta-analysis of photogrammetric studies. PLoS One 2015;10:e0134525. 10.1371/journal.pone.0134525 26247212 PMC4527668

[47] Zhu YJ, Zheng SW, Yang GS et al A novel method for 3d face symmetry reference plane based on weighted procrustes analysis algorithm. BMC Oral Health 2020;20:319. 10.1186/s12903-020-01311-3 33176780 PMC7659067

