# Supplementary material for: Automated assessment of 3D facial asymmetry: a systematic review
Source: Eur J Orthod. 2026 May 26;48(3):cjag012. doi: 10.1093/ejo/cjag012 (PMC13207581; doi:10.1093/ejo/cjag012)
Supplement: cjag012_Supplementary_Data [file cjag012_supplementary_data.zip › Supplementary Table S2.docx]

**Supplementary Table S2. Search strategy on each database**

| Database | Search strategy |
| --- | --- |
| Concept #1 facial asymmetry | |
| Pubmed | ((((facial asymmetry) OR (asymmetrical face)) OR (asymmetric face)) OR (craniofacial asymmetry) OR (facial asymmetry [MeSH Terms])) |
| Web of Science | ((TS = (facial asymmetry) OR TS = (asymmetrical face) OR TS = (asymmetric face) OR TS = (craniofacial asymmetry))) |
| EMBASE (via Ovid) | (facial asymmetry or asymmetrical face or asymmetric face or craniofacial asymmetry).af. |
| Medline (via Ovid) | (facial asymmetry or asymmetrical face or asymmetric face or craniofacial asymmetry).af. |
| Scopus | (TITLE-ABS-KEY (facial AND asymmetry) OR TITLE-ABS-KEY (asymmetrical AND face) OR TITLE-ABS-KEY (asymmetric AND face) OR TITLE-ABS-KEY (craniofacial AND asymmetry)) |
| Concept #2 three-dimensional facial scans | |
| Pubmed | ((((three-dimensional) OR (three dimensional)) OR (3D)) OR (imaging, three-dimensional [MeSH Terms])) |
| Web of Science | (((TS = (three-dimensional) OR TS = (three dimensional)) OR TS=(3D)) OR TS = (imaging, three-dimensional)) |
| EMBASE (via Ovid) | ((((three-dimensional) OR ("three dimensional")) OR (3D)) OR (exp "imaging, three-dimensional"/)) |
| Medline (via Ovid) | ((((three-dimensional) OR ("three dimensional")) OR (3D)) OR (exp "imaging, three-dimensional"/)) |
| Scopus | (((TITLE-ABS-KEY (three-dimensional) OR TITLE-ABS-KEY ("three dimensional")) OR TITLE-ABS-KEY (3D)) OR (INDEXTERMS ("imaging, three-dimensional"))) |
| Concept #3 automation | |
| Pubmed | (((((((((artificial intelligence) OR (AI)) OR (machine learning)) OR (deep learning)) OR (neural network*)) OR (automated)) OR (automatic)) OR (algorithm)) OR (Artificial Intelligence [MeSH Terms])) |
| Web of Science | ((TS = (artificial intelligence) OR TS = (AI) OR TS = (machine learning) OR TS = (deep learning) OR TS = (neural network*) OR TS = (automated) OR TS=(algorithm))) |
| EMBASE (via Ovid) | (artificial intelligence or AI or machine learning or deep learning or neural network* or automated or automatic or algorithm).af. |
| Medline (via Ovid) | (artificial intelligence or AI or machine learning or deep learning or neural network* or automated or automatic or algorithm).af. |
| Scopus | (TITLE-ABS-KEY (artificial intelligence) OR TITLE-ABS-KEY (AI) OR TITLE-ABS-KEY (machine learning) OR TITLE-ABS-KEY (deep learning) OR TITLE-ABS-KEY (neural network*) OR TITLE-ABS-KEY (automated) OR TITLE-ABS-KEY (automatic) OR TITLE-ABS-KEY (algorithm)) |
| Final search strategy | |
| Pubmed | ((((facial asymmetry) OR (asymmetrical face)) OR (asymmetric face)) OR (craniofacial asymmetry) OR (facial asymmetry [MeSH Terms])) AND ((((three-dimensional) OR (three dimensional)) OR (3D)) OR (imaging, three-dimensional [MeSH Terms])) AND (((((((((artificial intelligence) OR (AI)) OR (machine learning)) OR (deep learning)) OR (neural network*)) OR (automated)) OR (automatic)) OR (algorithm)) OR (Artificial Intelligence [MeSH Terms])) |
| Web of Science | ((TS = (facial asymmetry) OR TS = (asymmetrical face) OR TS = (asymmetric face) OR TS = (craniofacial asymmetry))) AND (((TS = (three-dimensional) OR TS = (three dimensional)) OR TS=(3D)) OR TS = (imaging, three-dimensional)) AND ((TS = (artificial intelligence) OR TS = (AI) OR TS = (machine learning) OR TS = (deep learning) OR TS = (neural network*) OR TS = (automated) OR TS=(algorithm))) |
| EMBASE (via Ovid) | (facial asymmetry or asymmetrical face or asymmetric face or craniofacial asymmetry).af. AND ((((three-dimensional) OR ("three dimensional")) OR (3D)) OR (exp "imaging, three-dimensional"/)) AND (artificial intelligence or AI or machine learning or deep learning or neural network* or automated or automatic or algorithm).af. |
| Medline (via Ovid) | (facial asymmetry or asymmetrical face or asymmetric face or craniofacial asymmetry).af. AND ((((three-dimensional) OR ("three dimensional")) OR (3D)) OR (exp "imaging, three-dimensional"/)) AND (artificial intelligence or AI or machine learning or deep learning or neural network* or automated or automatic or algorithm).af. |
| Scopus | (TITLE-ABS-KEY (facial AND asymmetry) OR TITLE-ABS-KEY (asymmetrical AND face) OR TITLE-ABS-KEY (asymmetric AND face) OR TITLE-ABS-KEY (craniofacial AND asymmetry)) AND (((TITLE-ABS-KEY (three-dimensional) OR TITLE-ABS-KEY ("three dimensional")) OR TITLE-ABS-KEY (3D)) OR (INDEXTERMS ("imaging, three-dimensional"))) AND (TITLE-ABS-KEY (artificial intelligence) OR TITLE-ABS-KEY (AI) OR TITLE-ABS-KEY (machine learning) OR TITLE-ABS-KEY (deep learning) OR TITLE-ABS-KEY (neural network*) OR TITLE-ABS-KEY (automated) OR TITLE-ABS-KEY (automatic) OR TITLE-ABS-KEY (algorithm)) |
