# Supplementary material for: Automated assessment of 3D facial asymmetry: a systematic review
Source: Eur J Orthod. 2026 May 26;48(3):cjag012. doi: 10.1093/ejo/cjag012 (PMC13207581; doi:10.1093/ejo/cjag012)
Supplement: cjag012_Supplementary_Data [file cjag012_supplementary_data.zip › Supplementary Table S3.docx]

**Supplementary Table S3. Studies excluded after full-text readings and reasons for exclusion (n = 37)**

| No. | Excluded study | Reason for exclusion |
| --- | --- | --- |
| 1 | Yamada et al., 2002 [1] | Wrong intervention: manual evaluation of facial asymmetry |
| 2 | Yu et al., 2009 [2] | Wrong intervention: lack of direct bilateral facial asymmetry assessment |
| 3 | Dyshkant et al., 2009 [3] | Wrong research filed: non-medically or biologically related field |
| 4 | Claes et al., 2011 [4] | The same method and research group with the included article [5] |
| 5 | Quan et al., 2012 [6] | Wrong intervention: manual evaluation of facial asymmetry |
| 6 | Walters et al., 2013 [7] | Wrong study design: case series |
| 7 | Berssenbrügge et al., 2014 [8] | Wrong outcome: lack of accuracy or reliability assessment |
| 8 | Modabber et al., 2014 [9] | Wrong outcome: lack of accuracy or reliability assessment |
| 9 | Desmedt et al., 2015 [10] | The same method and research group with the included article [11] |
| 10 | Djordjevic et al., 2016 [12] | Wrong intervention: manual evaluation of facial asymmetry |
| 11 | Wu et al., 2016 [13] | Wrong outcome: lack of accuracy or reliability assessment |
| 12 | Xiong et al., 2016 [14] | Wrong outcome: lack of accuracy or reliability assessment |
| 13 | Bockey et al., 2018 [15] | Wrong outcome: lack of accuracy or reliability assessment |
| 14 | Cho et al., 2018 [16] | Wrong outcome: lack of accuracy or reliability assessment |
| 15 | Sforza et al., 2018 [17] | Wrong intervention: lack of direct bilateral facial asymmetry assessment |
| 16 | Bouhjar et al., 2019 [18] | Wrong outcome: lack of accuracy or reliability assessment |
| 17 | Kordsmeyer et al., 2020 [19] | The same method and research group with the included article[5] |
| 18 | Lubarsch et al., 2020 [20] | Wrong outcome: lack of accuracy or reliability assessment |
| 19 | Lum et al., 2020 [21] | Wrong outcome: lack of accuracy or reliability assessment |
| 20 | Zhu et al., 2020 [22] | Wrong intervention: manual evaluation of facial asymmetry |
| 21 | Balestrini et al., 2021 [23] | Wrong outcome: lack of accuracy or reliability assessment |
| 22 | Fishman et al., 2021 [24] | Wrong intervention: lack of direct bilateral facial asymmetry assessment |
| 23 | Lo et al., 2021 [25] | Wrong outcome: lack of accuracy or reliability assessment |
| 24 | Alagha et al., 2022 [26] | Wrong outcome: lack of accuracy or reliability assessment |
| 25 | Fan et al., 2022 [27] | Wrong outcome: lack of accuracy or reliability assessment |
| 26 | Hansson et al., 2022 [28] | Wrong intervention: lack of direct bilateral facial asymmetry assessment |
| 27 | Lin et al., 2022 [29] | Wrong intervention: unknown 3D data types.  The same method and research group with the included article [30] |
| 28 | Ter Horst et al., 2022 [31] | Wrong outcome: lack of accuracy or reliability assessment |
| 29 | Zhu et al., 2022 [32] | Wrong intervention: lack of direct bilateral facial asymmetry assessment |
| 30 | Chien et al., 2023 [33] | Wrong outcome: lack of accuracy or reliability assessment |
| 31 | Patel et al., 2023 [34] | Wrong outcome: lack of accuracy or reliability assessment |
| 32 | Choi et al., 2024 [35] | Wrong outcome: lack of accuracy or reliability assessment |
| 33 | Prezelski et al., 2024 [36] | Wrong outcome: lack of accuracy or reliability assessment |
| 34 | Sukno et al., 2024 [37] | Wrong outcome: lack of accuracy or reliability assessment |
| 35 | Verhoeven et al., 2024 [38] | Wrong outcome: lack of accuracy or reliability assessment |
| 36 | Büchner et al., 2025 [39] | Wrong outcome: lack of accuracy or reliability assessment |
| 37 | Crins-de Koning et al., 2025 [40] | Wrong outcome: lack of accuracy or reliability assessment |

**References**

[1] T. Yamada, Y. Mori, K. Minami, K. Mishima, Y. Tsukamoto, Surgical results of primary lip repair using the triangular flap method for the treatment of complete unilateral cleft lip and palate: A three-dimensional study in infants to four-year-old children, CLEFT PALATE-CRANIOFACIAL JOURNAL 39(5) (2002) 497-502.<http://doi.org/10.1597/1545-1569(2002)039><0497:SROPLR>2.0.CO;2

[2] Z.Y. Yu, X.Z. Mu, S.Z. Feng, J.Y. Han, T.S. Chang, Flip-registration procedure of three-dimensional laser surface scanning images on quantitative evaluation of facial asymmetries, JOURNAL OF CRANIOFACIAL SURGERY 20(1) (2009) 157-160.<http://doi.org/10.1097/SCS.0b013e318191ce88>

[3] N. Dyshkant, L. Mestetskiy, Estimation of asymmetry in 3d face models, VISAPP 2009: PROCEEDINGS OF THE FOURTH INTERNATIONAL CONFERENCE ON COMPUTER VISION THEORY AND APPLICATIONS, VOL 1, 2009, pp. 402-405.

[4] P. Claes, M. Walters, D. Vandermeulen, J.G. Clement, Spatially-dense 3d facial asymmetry assessment in both typical and disordered growth, JOURNAL OF ANATOMY 219(4) (2011) 444-455.<http://doi.org/10.1111/j.1469-7580.2011.01411.x>

[5] O. Ekrami, P. Claes, J.D. White, A.A. Zaidi, M.D. Shriver, S. Van Dongen, Measuring asymmetry from high-density 3d surface scans: An application to human faces, PLOS ONE 13(12) (2018).<http://doi.org/10.1371/journal.pone.0207895>

[6] W. Quan, B.J. Matuszewski, L.K. Shark, Ieee, Facial asymmetry analysis based on 3-d dynamic scans, PROCEEDINGS 2012 IEEE INTERNATIONAL CONFERENCE ON SYSTEMS, MAN, AND CYBERNETICS (SMC), 2012, pp. 2676-2681.

[7] M. Walters, P. Claes, E. Kakulas, J.G. Clement, Robust and regional 3d facial asymmetry assessment in hemimandibular hyperplasia and hemimandibular elongation anomalies, INTERNATIONAL JOURNAL OF ORAL AND MAXILLOFACIAL SURGERY 42(1) (2013) 36-42.<http://doi.org/10.1016/j.ijom.2012.05.021>

[8] P. Berssenbrügge, N.F. Berlin, G. Kebeck, C. Runte, S. Jung, J. Kleinheinz, D. Dirksen, 2d and 3d analysis methods of facial asymmetry in comparison, JOURNAL OF CRANIO-MAXILLOFACIAL SURGERY 42(6) (2014) E327-E334.<http://doi.org/10.1016/j.jcms.2014.01.028>

[9] A. Modabber, M. Räsch, M. Ghassemi, M. Knobe, M. Gerressen, A. Ghassemi, M. Rana, F. Holzle, Noninvasive 3-dimensional evaluation of periorbital asymmetry in isolated unilateral orbital floor fractures, ORAL SURGERY ORAL MEDICINE ORAL PATHOLOGY ORAL RADIOLOGY 118(4) (2014) 392-399.<http://doi.org/10.1016/j.oooo.2014.05.010>

[10] D.J. Desmedt, T.J. Maal, M.A. Kuijpers, E.M. Bronkhorst, A.M. Kuijpers-Jagtman, P.S. Fudalej, Nasolabial symmetry and esthetics in cleft lip and palate: Analysis of 3d facial images, CLINICAL ORAL INVESTIGATIONS 19(8) (2015) 1833-1842.<http://doi.org/10.1007/s00784-015-1445-0>

[11] T.J. Verhoeven, C. Coppen, R. Barkhuysen, E.M. Bronkhorst, M.A.W. Merkx, S.J. Bergé, T.J.J. Maal, Three dimensional evaluation of facial asymmetry after mandibular reconstruction: Validation of a new method using stereophotogrammetry, INTERNATIONAL JOURNAL OF ORAL AND MAXILLOFACIAL SURGERY 42(1) (2013) 19-25.<http://doi.org/10.1016/j.ijom.2012.05.036>

[12] J. Djordjevic, A.I. Zhurov, S. Richmond, V. Consortium, Genetic and environmental contributions to facial morphological variation: A 3d population-based twin study, PLOS ONE 11(9) (2016).<http://doi.org/10.1371/journal.pone.0162250>

[13] J. Wu, S. Liang, L. Shapiro, R. Tse, Measuring symmetry in children with cleft lip. Part 2: Quantification of nasolabial symmetry before and after cleft lip repair, CLEFT PALATE-CRANIOFACIAL JOURNAL 53(6) (2016) 705-713.<http://doi.org/10.1597/15-220>

[14] Y.X. Xiong, Y.J. Zhao, H.F. Yang, Y.C. Sun, Y. Wang, Comparison between interactive closest point and procrustes analysis for determining the median sagittal plane of three-dimensional facial data, JOURNAL OF CRANIOFACIAL SURGERY 27(2) (2016) 441-444.<http://doi.org/10.1097/SCS.0000000000002376>

[15] S. Bockey, P. Berssenbrügge, D. Dirksen, K. Wermker, M. Klein, C. Runte, Computer-aided design of facial prostheses by means of 3d-data acquisition and following symmetry analysis, JOURNAL OF CRANIO-MAXILLOFACIAL SURGERY 46(8) (2018) 1320-1328.<http://doi.org/10.1016/j.jcms.2018.05.020>

[16] M.J. Cho, R.R. Hallac, J. Ramesh, J.R. Seaward, N.V. Hermann, T.A. Darvann, A. Lipira, A.A. Kane, Quantifying normal craniofacial form and baseline craniofacial asymmetry in the pediatric population, PLASTIC AND RECONSTRUCTIVE SURGERY 141(3) (2018) 380E-387E.<http://doi.org/10.1097/PRS.0000000000004114>

[17] C. Sforza, E. Ulaj, D.M. Gibelli, F. Allevi, V. Pucciarelli, F. Tarabbia, D. Ciprandi, G.D. Orabona, C. Dolci, F. Biglioli, Three-dimensional superimposition for patients with facial palsy: An innovative method for assessing the success of facial reanimation procedures, BRITISH JOURNAL OF ORAL & MAXILLOFACIAL SURGERY 56(1) (2018) 3-7.<http://doi.org/10.1016/j.bjoms.2017.11.015>

[18] N. Ben Bouhjar, J. Kleinheinz, D. Dirksen, P. Berssenbrügge, C. Runte, K. Wermker, Facial and midfacial symmetry in cleft patients: Comparison to non-cleft children and influence of the primary treatment concept, JOURNAL OF CRANIO-MAXILLOFACIAL SURGERY 47(5) (2019) 741-749.<http://doi.org/10.1016/j.jcms.2019.01.041>

[19] T.L. Kordsmeyer, Y.T.K. Thies, O. Ekrami, J. Stern, C. Schild, C. Spoiala, P. Claes, S. Van Dongen, L. Penke, No evidence for an association between facial fluctuating asymmetry and vocal attractiveness in men or women, EVOLUTIONARY HUMAN SCIENCES 2 (2020).<http://doi.org/10.1017/ehs.2020.36>

[20] M. Blanck-Lubarsch, D. Dirksen, R. Feldmann, C. Sauerland, C. Kirschneck, A. Hohoff, Asymmetry-index and orthodontic facial analysis of children with foetal alcohol syndrome using 3d-facial scans, PEDIATRIC RESEARCH 88(2) (2020) 243-249.<http://doi.org/10.1038/s41390-019-0559-5>

[21] V. Lum, M.S. Goonewardene, A. Mian, P. Eastwood, Three-dimensional assessment of facial asymmetry using dense correspondence symmetry, and midline analysis, AMERICAN JOURNAL OF ORTHODONTICS AND DENTOFACIAL ORTHOPEDICS 158(1) (2020) 134-146.<http://doi.org/10.1016/j.ajodo.2019.12.014>

[22] Y.J. Zhu, S.W. Zheng, G.S. Yang, X.L. Fu, N. Xiao, A.N. Wen, Y. Wang, Y.J. Zhao, A novel method for 3d face symmetry reference plane based on weighted procrustes analysis algorithm, BMC ORAL HEALTH 20(1) (2020).<http://doi.org/10.1186/s12903-020-01311-3>

[23] S. Balestrini, S.M. Lopez, K. Chinthapalli, N. Sargsyan, R. Demurtas, S. Vos, A. Altmann, M. Suttie, P. Hammond, S.M. Sisodiya, Increased facial asymmetry in focal epilepsies associated with unilateral lesions, BRAIN COMMUNICATIONS 3(2) (2021).<http://doi.org/10.1093/braincomms/fcab068>

[24] Z. Fishman, C.M. Whyne, A. Hope, J.A. Fialkov, Modeling and measuring average nasal asymmetry by dorsum midline and nose tip lateral deviation, JOURNAL OF PLASTIC RECONSTRUCTIVE AND AESTHETIC SURGERY 74(4) (2021) 857-865.<http://doi.org/10.1016/j.bjps.2020.10.017>

[25] L.J. Lo, C.T. Yang, C.T. Ho, C.H. Liao, H.H. Lin, Automatic assessment of 3-dimensional facial soft tissue symmetry before and after orthognathic surgery using a machine learning model a preliminary experience, ANNALS OF PLASTIC SURGERY 86(3S) (2021) S224-S228.<http://doi.org/10.1097/SAP.0000000000002687>

[26] M.A. Alagha, A. Ayoub, S. Morley, X.Y. Ju, Objective grading facial paralysis severity using a dynamic 3d stereo photogrammetry imaging system, OPTICS AND LASERS IN ENGINEERING 150 (2022).<http://doi.org/10.1016/j.optlaseng.2021.106876>

[27] Y. Fan, W. He, G. Chen, G.Y. Song, H. Matthews, P. Claes, R.P. Jiang, T.M. Xu, Facial asymmetry assessment in skeletal class iii patients with spatially-dense geometric morphometrics, EUROPEAN JOURNAL OF ORTHODONTICS 44(2) (2022) 155-162.<http://doi.org/10.1093/ejo/cjab034>

[28] S. Hansson, E. Östlund, F. Bazargani, The vectra m3 3-dimensional digital stereophotogrammetry system: A reliable technique for detecting chin asymmetry, IMAGING SCIENCE IN DENTISTRY 52(1) (2022) 43-51.<http://doi.org/10.5624/isd.20210168>

[29] H.H. Lin, T. Zhang, Y.C. Wang, C.T. Yang, L.J. Lo, C.H. Liao, S.K. Kuang, A system for quantifying facial symmetry from 3d contour maps based on transfer learning and fast r-cnn, JOURNAL OF SUPERCOMPUTING 78(14) (2022) 15953-15973.<http://doi.org/10.1007/s11227-022-04502-7>

[30] H.H. Lin, L.J. Lo, W.C. Chiang, A novel assessment technique for the degree of facial symmetry before and after orthognathic surgery based on three-dimensional contour features using deep learning algorithms, ACM International Conference Proceeding Series, 2019, pp. 170-173.

[31] R. Ter Horst, T.J.J. Maal, M.J.J. de Koning, J.S. Mertens, E.J.H. Schatorjé, E.P. Hoppenreijs, M.M.B. Seyger, 3d stereophotogrammetry in children and adolescents with scleroderma en coup de sabre/parry-romberg syndrome: Description of a novel method for monitoring disease progression, Skin Health Dis 2(3) (2022) e132.<http://doi.org/10.1002/ski2.132>

[32] Y.J. Zhu, X.L. Fu, L. Zhang, S.W. Zheng, A.N. Wen, N. Xiao, Y. Wang, Y.J. Zhao, A mathematical algorithm of the facial symmetry plane: Application to mandibular deformity 3d facial data, JOURNAL OF ANATOMY 240(3) (2022) 556-566.<http://doi.org/10.1111/joa.13564>

[33] C.F. Chien, J.L. Sung, C.P. Wang, C.W. Yen, Y.H. Yang, Analyzing facial asymmetry in alzheimer's dementia using image-based technology, BIOMEDICINES 11(10) (2023).<http://doi.org/10.3390/biomedicines11102802>

[34] Y. Patel, I. Sharp, L. Enocson, B.S. Khambay, An innovative analysis of nasolabial dynamics of surgically managed adult patients with unilateral cleft lip and palate using 3d facial motion capture, JOURNAL OF PLASTIC RECONSTRUCTIVE AND AESTHETIC SURGERY 85 (2023) 287-298.<http://doi.org/10.1016/j.bjps.2023.07.007>

[35] T.M. Choi, X.J. Liu, T. Abdel-Alim, M.L. van Veelen, I.M.J. Mathijssen, E.B. Wolvius, G.V. Roshchupkin, Automated three-dimensional analysis of facial asymmetry in patients with syndromic coronal synostosis: A retrospective study, JOURNAL OF CRANIO-MAXILLOFACIAL SURGERY 52(1) (2024) 48-54.<http://doi.org/10.1016/j.jcms.2023.11.006>

[36] K. Prezelski, J.A.T. Cheng, R.R. Hallac, Dynamic three-dimensional facial topography in pediatric facial palsy: Understanding asymmetrical facial contours, JOURNAL OF PLASTIC RECONSTRUCTIVE AND AESTHETIC SURGERY 99 (2024) 494-501.<http://doi.org/10.1016/j.bjps.2024.10.024>

[37] F.M. Sukno, B.D. Kelly, A. Lane, S. Katina, M.A. Rojas, P.F. Whelan, J.L. Waddington, Loss of normal facial asymmetry in schizophrenia and bipolar disorder: Implications for development of brain asymmetry in psychotic illness, PSYCHIATRY RESEARCH 342 (2024).<http://doi.org/10.1016/j.psychres.2024.116213>

[38] T.J. Verhoeven, S. Vinayahalingam, G. Claeys, T. Xi, S.J. Berge, T.J.J. Maal, Does facial asymmetry vary between subjects of different age groups? A 3d stereophotogrammetry analysis, JOURNAL OF CRANIO-MAXILLOFACIAL SURGERY 52(7) (2024) 829-834.<http://doi.org/10.1016/j.jcms.2024.04.003>

[39] T. Büchner, S. Sickert, G.F. Volk, O. Guntinas-Lichius, J. Denzler, Assessing 3d volumetric asymmetry in facial palsy patients via advanced multi-view landmarks and radial curves, MACHINE VISION AND APPLICATIONS 36(1) (2025).<http://doi.org/10.1007/s00138-024-01616-1>

[40] M. Crins-de Koning, R. Bruggink, M. Nienhuijs, T. Wagner, E.M. Bronkhorst, E.M. Ongkosuwito, Three-dimensional analysis of facial morphology in nine-year-old children with different unilateral orofacial clefts compared to normative data, PEERJ 13 (2025).<http://doi.org/10.7717/peerj.18739>
