# Supplementary material for: Automated assessment of 3D facial asymmetry: a systematic review
Source: Eur J Orthod. 2026 May 26;48(3):cjag012. doi: 10.1093/ejo/cjag012 (PMC13207581; doi:10.1093/ejo/cjag012)
Supplement: cjag012_Supplementary_Data [file cjag012_supplementary_data.zip › Supplementary Table S4.docx]

**Supplementary Table S4. tailored QUADAS-2​**

**Domain 1: Patient Selection​​**

Signaling Questions:​​

1. *Was a consecutive or random sample of subjects enrolled?​*

​

Yes: Explicitly states consecutive/random recruitment from a defined clinical population.

No: Convenience sampling, case-control design, or pre-selected groups (e.g., symmetrical vs. asymmetrical cohorts).

Unclear: Recruitment methodology inadequately described.

Risk of Bias:​​

Low: All answers = Yes.

High: Any answer = No.

Unclear: Insufficient reporting of enrollment criteria.

Applicability:​​

Low concern: Subjects/sampling units represent clinically relevant facial asymmetry populations with detailed demographics.

High concern: Non-representative sample (e.g., artificial models, extreme phenotypes without clinical generalizability).

Unclear: Insufficient description of population characteristics to assess representativeness. More than one of the three indicators including sex, age, and ethnicity is not clearly reported.

**Domain 2: Index Test (Automated 3D Analysis)**

Signaling Questions:

*1. Was the automated asymmetry analysis protocol sufficiently detailed?​*

Yes: Full technical specifications provided (e.g., software, algorithms, preprocessing steps).

No: Incomplete description of methodology (e.g., missing parameters, proprietary tools without validation).

Unclear: Partial technical details reported.

Risk of Bias:​​

Low: All answers = Yes.

High: Any answer = No.

Unclear: Insufficient reporting of enrollment criteria.

Applicability:​​

Low concern: Index test aligns with clinical diagnostic workflows (e.g., surgical planning, orthodontic evaluation).

High concern: Purely experimental metrics lacking clinical relevance.

Unclear: Clinical relevance of asymmetry metrics ambiguously described (e.g., thresholds mentioned without linking to treatment decisions).

**Domain 3: Reference Standard**

Signaling Questions:​​

1. *Does the reference standard reliably quantify ground-truth asymmetry?​*

Yes: Uses validated clinical measures (e.g., expert panels, synthetic asymmetry pattern).

No: unvalidated methods(e.g., landmark-based method) or lack of validation

Unclear: Reference standard ambiguously described.

1. *Were reference standard assessors blinded to index test results?​*

Yes: Independent evaluation with blinding protocols (e.g., temporal separation, anonymized data).

No: Assessors aware of automated analysis outcomes.

Unclear: Blinding procedures not specified.

​​

Risk of Bias:​​

Low: All answers = Yes.

High: Any answer = No.

Unclear: Insufficient reporting of reference methodology.

Applicability:​​

Low concern: Reference standard matches clinical gold standards (e.g., multi-expert consensus, validated imaging protocols, synthetic datasets).

High concern: poorly validated comparators (e.g., landmark-based method) or lack of validation

Unclear: Incomplete reporting of reference standard validation (e.g., expert qualifications unstated, inter-rater reliability not quantified).

**Domain 4: Flow and Timing**

Signaling Questions: ​

1. *Was a uniform reference standard applied to all subjects?​*

Yes: All participants underwent identical reference evaluations (e.g., same imaging modality/expert panel).

No: Differential standards used (e.g., radiographs for some, clinical exams for others).

1. *Was the automated method validated for reliability?​​*

Yes: Reports test-retest reliability, intraclass correlation coefficients, or error margins.

No: No validation metrics provided.

Risk of Bias:

Low: All answers = Yes.

High: Any answer = No.

Unclear: Flow diagram missing or incomplete.
