# Supplementary material for: Automated assessment of 3D facial asymmetry: a systematic review
Source: Eur J Orthod. 2026 May 26;48(3):cjag012. doi: 10.1093/ejo/cjag012 (PMC13207581; doi:10.1093/ejo/cjag012)
Supplement: cjag012_Supplementary_Data [file cjag012_supplementary_data.zip › Supplementary Table S5.docx]

**Supplementary Table S5. Risk of bias of the included studies in this systematic review with supporting reasons**

| No. | Author/year | Risk of bias | | | | Applicability concerns | | |
| --- | --- | --- | --- | --- | --- | --- | --- | --- |
|  |  | Patient selection | Index test | Reference standard | Flow and timing | Patient selection | Index test | Reference standard |
| 1 | Darvann et al./2011 [14] | Low risk: Consecutive inclusion | High risk: Lack of details about image preprocessing, initial alignment, details about algorithm, and purpose of mid-sagittal plane | High risk: Reference standard (landmark-based method) is subjective and error-prone | High risk: Lack of reliability metrics undermines reproducibility | Low concern: Clinically relevant facial asymmetry populations with detailed demographics | Low concern: Metrics align with clinical workflows | High concern: Reference standard (landmark-based method) is subjective and error-prone |
| 2 | Verhoeven et al. /2013 [15] | High risk: Not consecutive/random inclusion | Low risk: Full technical specifications provided | High risk: Reliance on unvalidated clinical judgments instead of gold standards | High risk: Lack of uniform reference standard | Low concern: Clinically relevant facial asymmetry populations with detailed demographics | Low concern: Metrics align with clinical workflows | High concern: Reliance on unvalidated clinical judgments instead of gold standards |
| 3 | Alqattan et al./2015 [16] | High risk: Not consecutive/random inclusion | High risk: Lack of details about image preprocessing, initial alignment, and refection plane | High risk: Reference standard (landmark-based method) is subjective and error-prone | High risk: Lack of uniform reference standard | Low concern: Clinically relevant facial asymmetry populations with detailed demographics | Low concern: Metrics align with clinical workflows | High concern: Reference standard (landmark-based method) is subjective and error-prone |
| 4 | Patel A et al./2015 [17] | Unclear risk: Insufficient reporting of enrollment criteria | High risk: Lack of details about algorithms and landmarking for region division | High risk: Reliance on unvalidated clinical judgments instead of gold standards | High risk: Lack of uniform reference standard | Unclear concern: Insufficient demographic details | Low concern: Metrics align with clinical workflows | High concern: Reliance on unvalidated clinical judgments instead of gold standards |
| 5 | Sukno et al./2015 [18] | High risk: Not consecutive/random inclusion | Low risk: Full technical specifications provided | Low risk: Quantitative ground truth with controlled deformations | High risk: Lack of reliability metrics undermines reproducibility | Unclear concern: Insufficient demographic details | Low concern: Metrics align with clinical workflows | Low concern: Synthetic asymmetry patterns emulate clinical dysmorphology |
| 6 | Liang et al./2017 [19] | Low risk: Consecutive inclusion | Low risk: Full technical specifications provided | High risk: The reference standard ("expert-ranked cleft severity") relies on a single surgeon’s unvalidated ranking | High risk: Lack of reliability metrics undermines reproducibility | Low concern: Clinically relevant facial asymmetry populations with detailed demographics | Low concern: Metrics align with clinical workflows | High concern: Expert ranking is clinically relevant but poorly validated (no inter-rater reliability or expert qualification details). |
| 7 | Al-Rudainy et al. /2018 [20] | High risk: Not consecutive/random inclusion | Low risk: Full technical specifications provided | High risk: Absence of a validated reference standard prevents verification of the automated method's accuracy | High risk: Lack of uniform reference standard | Low concern: Clinically relevant facial asymmetry populations with detailed demographics | Low concern: Metrics align with clinical workflows | High concern: Without comparison to a clinical gold standard (e.g., multi-rater consensus), the clinical relevance of the asymmetry metrics remains unverified. |
| 8 | Ekrami O et al./2018 [21] | Unclear risk: Insufficient reporting of enrollment criteria | Low risk: Full technical specifications provided | Low risk: Quantitative ground truth with controlled deformations | High risk: Lack of reliability metrics undermines reproducibility | Low concern: Clinically relevant facial asymmetry populations with detailed demographics | Low concern: Metrics align with clinical workflows | Low concern: Synthetic asymmetry patterns emulate clinical dysmorphology |
| 9 | Lin et al. /2019 [22] | High risk: Not consecutive/random inclusion | High risk: Lack of details about construction of mid-sagittal plane and contour maps | High risk: Unvalidated reference standard (50 untrained human raters using a subjective 10-point scale) | High risk: Lack of reliability metrics undermines reproducibility | Low concern: Clinically relevant facial asymmetry populations with detailed demographics | Low concern: Metrics align with clinical workflows | High concern: (Subjective human ratings are not a clinical gold standard; lack of expert validation limits relevance) |
| 10 | Bernini et al. /2020 [23] | Low risk: Consecutive inclusion | High risk: Lack of details about algorithm, reflection plane, and landmarking for region division | High risk: Absence of a validated reference standard prevents verification of the automated method's accuracy | High risk: Lack of uniform reference standard | Low concern: Clinically relevant facial asymmetry populations with detailed demographics | Low concern: Metrics align with clinical workflows | High concern: Without comparison to a clinical gold standard (e.g., multi-rater consensus), the clinical relevance of the asymmetry metrics remains unverified |
| 11 | Hallac et al./ 2020 [24] | High risk: Not consecutive/random inclusion | Low risk: Full technical specifications provided | High risk: Absence of a validated reference standard prevents verification of the automated method's accuracy | High risk: Lack of uniform reference standard | Low concern: Clinically relevant facial asymmetry populations with detailed demographics | Low concern: Metrics align with clinical workflows | High concern: Without comparison to a clinical gold standard (e.g., multi-rater consensus), the clinical relevance of the asymmetry metrics remains unverified |
| 12 | Gkantidis et al. /2023 [25] | Low risk: Random inclusion | Low risk: Full technical specifications provided | High risk: Absence of a validated reference standard prevents verification of the automated method's accuracy | High risk: Lack of uniform reference standard | Low concern: Clinically relevant facial asymmetry populations with detailed demographics | Low concern: Metrics align with clinical workflows | High concern: Without comparison to a clinical gold standard (e.g., multi-rater consensus), the clinical relevance of the asymmetry metrics remains unverified |
| 13 | Zhao et al./2023 [26] | Low risk: Random inclusion | Low risk: Full technical specifications provided | High risk: Absence of a validated reference standard prevents verification of the automated method's accuracy | High risk: Lack of uniform reference standard | Low concern: Clinically relevant facial asymmetry populations with detailed demographics | Low concern: Metrics align with clinical workflows | High concern: Without comparison to a clinical gold standard (e.g., multi-rater consensus), the clinical relevance of the asymmetry metrics remains unverified |
| 14 | Yang et al./2025 [27] | Low risk: Random inclusion | Low risk: Full technical specifications provided | High risk: Unvalidated reference method (original-mirror method) | Low risk: All subjects underwent identical reference (mirroring analysis) and index (wireframe) tests; test-retest reliability was reported. | Low concern: Clinically relevant facial asymmetry populations with detailed demographics | Low concern: Metrics align with clinical workflows | High concern: Without comparison to a clinical gold standard (e.g., multi-rater consensus), the clinical relevance of the asymmetry metrics remains unverified |
