# Supplementary material for: Automated assessment of 3D facial asymmetry: a systematic review
Source: Eur J Orthod. 2026 May 26;48(3):cjag012. doi: 10.1093/ejo/cjag012 (PMC13207581; doi:10.1093/ejo/cjag012)
Supplement: cjag012_Supplementary_Data [file cjag012_supplementary_data.zip › Supplementary Table S6.docx]

**Supplementary Table S6. Detailed demographic characteristics of the included studies**

| No. | Author/year | Sample source | Sample size | Sex (M/F) | Mean age/age range | Ethnicity | Random or Consecutive inclusion |
| --- | --- | --- | --- | --- | --- | --- | --- |
| 1 | Darvann et al./2011 [14] | JIA patients with unilateral TMJ involvement and Healthy children | 44 |  |  |  |  |
|  |  | Patients group | 22 | 3M/19F | 12.2 years/ 4–17 years | Danish Caucasians | Consecutive |
|  |  | Control group | 22 | Gender-matched | Age-matched | Danish Caucasians | NR |
| 2 | Verhoeven et al. /2013 [15] | Patients who underwent mandibular reconstruction and healthy volunteers | 39 |  |  |  |  |
|  |  | Patients group | 15 | 10M/5F | 54 years/ 15–74 years | NR | NR |
|  |  | Control group | 24 | Gender-matched | Age-matched | NR | NR |
| 3 | Alqattan et al./2015 [16] | Orthodontic patients with marked facial asymmetry and students/staff of Cardiff Dental Hospital | 88 |  |  |  |  |
|  |  | Patients group | 3 | 1M/2F | 33 years/19-42 years | British Caucasians | NR |
|  |  | Control group | 85 | 29M/56F | Males: 23.9 years/ 19–44 years; females: 28.1 years/ 19–54 years | NR | NR |
| 4 | Patel A et al./2015 [17] | Orthodontic patients | 58 | NR | ≥20 years old | NR | NR |
| 5 | Sukno et al./2015 [18] | Healthy volunteers | 100 | NR | NR | NR | NR |
| 6 | Liang et al./2017 [19] | UCL patients and control group from Seattle Children’s Hospital and normal reference from 3D Facial Norms Database | 63 |  |  |  |  |
|  |  | UCL Infants​ | 30 | 17M/13F | 5.8 months | NR | Consecutive |
|  |  | UCL Children | 27 | 17M/10F | 10.6 years | NR | Consecutive |
|  |  | Control Infants | 3 | 1M/2F | 6.2 months | NR | NR |
|  |  | Control Children | 3 | 1M/2F | 10.1 years | NR | NR |
|  |  | Normal reference | 2279 | NR | NR | Caucasian | NR |
| 7 | Al-Rudainy et al. /2018 [20] | UCLP infants from the Royal Hospital for Sick Children, Edinburgh, UK | 26 | NR | Pre-surgery: 3.6 months; post-surgery: 8.2 months | Caucasian | NR |
| 8 | Ekrami O et al./2018 [21] | Penn State University dataset | 430 |  |  |  | NR |
|  |  | Anthropometric mask construction | 400 | NR | 18-25 years | Western Australian |  |
|  |  | Validation dataset | 30 | NR | 18-30 years | Mixed ancestries |  |
| 9 | Lin et al. /2019 [22] | Patients who underwent orthognathic surgery from Chang Gung Memorial Hospital in Taiwan and normal population for training/validation/test dataset | 195 |  |  |  |  |
|  |  | Patients group | 100 | 56M/44F | 28.7 years/19-34 years | NR | NR |
|  |  | Normal population | 500 | NR | NR | NR | NR |
| 10 | Bernini et al. /2020 [23] | JIA patients from a university clinic and children’s hospital | 76 | 26M/50F | 11.7 years/ 6.3–17.9 years | NR | Consecutive |
| 11 | Hallac et al./ 2020 [24] | Healthy pediatric volunteers | 36 | 23M/13F | 8-12 years | NR | NR |
| 12 | Gkantidis et al. /2023 [25] | Orthodontic patients from the University of Bern, Switzerland | 20 |  |  |  |  |
|  |  | Children group | 10 | NR | 8-12 years | NR | Random |
|  |  | Adults group | 10 | NR | 20-40 years | NR | Random |
| 13 | Zhao et al./2023 [26] | Patients from the Stomatology Hospital of Xian Jiaotong University, China | 270 | 135M/135M | 18–30 years | Chinese | Random |
| 14 | Yang et al./2025 [27] | Orthodontic patients from Peking University Stomatological Hospital | 24 | 14M/10F | 12–32 years | NR | Random |

Notes: M = male, F = female, JIA = juvenile idiopathic arthritis, TMJ = temporomandibular joint, NR = Not reported, UCL = unilateral cleft lip, 3D = three-dimensional, UCLP = unilateral cleft lip and palate.
