# Supplementary material for: Automated assessment of 3D facial asymmetry: a systematic review
Source: Eur J Orthod. 2026 May 26;48(3):cjag012. doi: 10.1093/ejo/cjag012 (PMC13207581; doi:10.1093/ejo/cjag012)
Supplement: cjag012_Supplementary_Data [file cjag012_supplementary_data.zip › Supplementary Table S7.docx]

**Supplementary Table S7. Detailed methodological characteristics of the included studies**

| No. | Author/year | Software used | Algorithm used | MSP | MSP construction | Reflection plane | Global/regional analysis | Landmarking for region division | Asymmetry assessment | Validity assessment method | Validity assessment result | Reliability assessment method | Reliability assessment result |
| --- | --- | --- | --- | --- | --- | --- | --- | --- | --- | --- | --- | --- | --- |
| 1 | Darvann et al./2011 [14] | Custom algorithms | B-spline-based non-rigid registration | Defined by 3 landmarks | Manual | The same plane with MSP | Global and regional | NR | 1. Individual asymmetry: distance between original and mirrored surface 2. Inter-individual asymmetry: distance between the symmetric atlas surface and the symmetric version of each patient’s face | r with manual landmark-based method | r = 0.92 | NR | NR |
| 2 | Verhoeven et al. /2013 [15] | 3dMD Patient™ for image preprocessing, Maxilim® for manual landmarking and surface registration, MATLAB® for calculation of asymmetry | ICP algorithm (in-house algorithm of Maxilim®) | Defined by 4 landmarks | Manual | The same plane with MSP | Global and regional | Manual | MAD between original and mirrored surface | NR | NR | Two observers and repetition | Inter-observer difference: 0.04 mm; Intra-observer difference: 0.02 mm |
| 3 | Alqattan et al./2015 [16] | Rapidform 2006 for asymmetry analysis | ICP algorithm (in-house algorithm of Rapidform 2006) | MSP-independent | MSP-independent | NR | Global and regional | Manual | 1. Distance between original and mirrored surface  2. The percentage of match between the two facial surfaces to within a threshold (0.5mm) | NR | NR | Repetition | Intra-observer difference: within 0.005mm |
| 4 | Patel A et al./2015 [17] | 3dMDpatient for image preprocessing, 3dMDvultus for asymmetry analysis | Levenberg-Marquardt algorithm | MSP-independent | MSP-independent | Arbitrary plane outside of the face | Global and regional | NR | RMS between original and mirrored surfaces | NR | NR | Repetition | Intra-observer ICC: generally high (values not fully reported) |
| 5 | Sukno et al./2015 [18] | Custom algorithms | Template-mapping + HM-DW-LMedS algorithm | NR | NR | N/A | Global | N/A | Distance between original and mirrored surface | r between estimated asymmetry and 25 synthetic asymmetry patterns | r > 0.9 for most patterns | NR | NR |
| 6 | Liang et al./2017 [19] | Face++ for landmark detection, custom algorithms for asymmetry analysis | NR | Defined by 4 landmarks | Automated | N/A | Regional | Automated | BAD in different depth | r between BAD and expert-ranked cleft severity | r = 0.70 | NR | NR |
| 7 | Al-Rudainy et al. /2018 [20] | VRMesh for image preprocessing and asymmetry analysis | ICP algorithm with 0.5 mm tolerance (in-house algorithm of VRMesh) | MSP-independent | MSP-independent | A reflection  plane outside the face | Global and regional | Manual | The 90% of the absolute linear distances between original and mirrored surfaces | NR | NR | Repetition | Intra-observer difference: no statistical significance (p > 0.05) |
| 8 | Ekrami O et al./2018 [21] | Custom algorithms | Template-mapping and robust PA | MSP-independent | MSP-independent | N/A | Global | N/A | DA: MSD between average surface of the population and average surface of original and mirrored one  FA: MSD between original and mirrored surface - DA | r^2^ between true simulated FA values and the estimated FA values | r^2 =^ 0.99 | NR | NR |
| 9 | Lin et al. /2019 [22] | Custom algorithms | CNN | NR | NR | N/A | Global | N/A | Overlap feature of bilateral contour line | Comparison with average ratings from 50 diverse reviewers using a 10-point symmetry scale | 78.85% accuracy on test dataset; 98.63% within 1 degree error | NR | NR |
| 10 | Bernini et al. /2020 [23] | 3d Slicer for image preprocessing and asymmetry analysis | ICP algorithm (in-house algorithm of 3d Slicer) | MSP-independent | MSP-independent | NR | Regional | NR | HD between original and mirrored surfaces for chin area | NR | NR | Repetition | Intra-observer ICC: 0.95 for max HAD and 0.81 for mean HAD |
| 11 | Hallac et al./ 2020 [24] | Di3D for template registration; Di4DTrack for semi-landmarks tracking, MATLAB for calculation of asymmetry | Template-mapping | MSP-independent | MSP-independent | N/A | Regional | NR | The sum of bilateral difference of semi-landmarks displacement | NR | NR | Two observers | CCC = 0.98 |
| 12 | Gkantidis et al. /2023 [25] | Viewbox 4 software for image preprocessing and asymmetry analysis | ICP algorithm (in-house algorithm of Viewbox 4 software) | Derived from ICP algorithm | Landmark-independent | NR | Regional | NR | MAD between original and mirrored surfaces for chin area | NR | NR | Repetition | Intra-observer difference: within 0.01 mm |
| 13 | Zhao et al./2023 [26] | Geomagic Wrap for asymmetry analysis | ICP algorithm (in-house algorithm of Geomagic Wrap) | Defined by 3 landmarks | Manual | NR | Global and regional | Manual | RMS between original and mirrored surfaces | NR | NR | Two observers and repetition | Inter-observer ICC: 0.916 (p < 0.001) Intra-observer ICC: 0.945 (p < 0.001) |
| 14 | Yang et al./2025 [27] | Matlab and Meshmonk toolbox for landmark detection and asymmetry analysis | Template-mapping + robust PA | MSP-independent | MSP-independent | N/A | Regional | N/A | \|Angle_R_−Angle_L_\|/Angle_L_ × 100% | Classification consistency rates by comparison with original-mirror alignment method | Consistency rates: 87.5–100% for different regions | Repetition | Kappa value: 0.664–1.00 |

Notes: MSP = mid-saggital plane, NR = Not reported, r = correlation coefficients, ICP = Iterative Closest Point, MAD = mean absolute distance, RMS = root mean square, N/A = Not applicable, ICC = intraclass correlation coefficient, HM-DW-LMedS = Hemispheres-Distance-weighted Least Median of Squares, BAD = bilateral area difference, PA = Procrustes analysis, DA = directional asymmetry, MSD = mean signed distance, FA = fluctuating asymmetry, r^2^ = coefficient of determination, CNN = convolutional neural network, HD = Hausdorff distance, CCC = Lin’s concordance correlation coefficient, 4D = four-dimensional.
